# Serum levels of adipokines and cytokines in psoriasis patients: a systematic review and meta-analysis

**DOI:** 10.18632/oncotarget.22260

**Published:** 2017-11-01

**Authors:** Fan Bai, Wen Zheng, Yan Dong, Juan Wang, Malgorzata A. Garstka, Ruilian Li, Jingang An, Huiqun Ma

**Affiliations:** ^1^ The Second Affiliated Hospital of Xi'an Jiaotong University, Xi’an, China

**Keywords:** psoriasis, serum, adipokine, cytokine, meta-analysis

## Abstract

**Purpose:**

To evaluate the association of serum levels of adipokines and cytokines with psoriasis.

**Materials and Methods:**

A comprehensive literature search was performed in PubMed, ScienceDirect and Web of Science for the available relevant studies published before December 1, 2016. Differences in serum marker levels between patients and controls were pooled as standardized mean differences (SMDs) with 95% confidence interval to combine the effect estimations. We also conducted stratified analysis, meta-regression analysis and sensitivity analysis.

**Results:**

Sixty-three studies containing 2876 psoriasis patients and 2237 healthy controls were included in this meta-analysis. The pooled serum levels of TNF-α, IFN-γ, IL-2, IL-6, IL-8, IL-18, IL-22, chemerin, lipocalin-2, resistin, sE-selectin, fibrinogen and C3 were higher in psoriasis patients compared with healthy controls (all *P* < 0.05). In contrast, adiponectin levels were lower. Serum levels of IL-1β, IL-4, IL-10, IL-12, IL-17, IL-21, IL-23, visfatin and omentin were not significantly different between psoriasis patients and controls (all *P* > 0.05). However, increased serum levels of IL-17 correlated with psoriasis in men. For other biomarkers, age, gender and psoriasis area and severity index did not explain the differences in effect size between the studies.

**Conclusions:**

Serum levels of TNF-α, IFN-γ, IL-2, IL-6, IL-8, IL-18, IL-22, chemerin, lipocalin-2, resistin, sE-selectin, fibrinogen, complement 3, and adiponectin correlate with psoriasis and can be used as potential biomarkers for psoriasis and response to the treatment. Future studies are needed to identify additional players involved in the pathogenesis of psoriasis and to fully decipher the underlying mechanism.

## INTRODUCTION

Psoriasis is a serious, chronic, immune-mediated, hyperproliferative, and inflammatory skin disease of varying severity, which may induce itchy or painful lesions and negatively impact quality of life [1, 2]. The prevalence of this disease ranges from 0.51% to 11.43% in different countries [3].

The genetic, immunological and environmental factors contribute to the pathogenesis of psoriasis. However, its precise etiology is not yet fully elucidated. The pathological mechanism in psoriasis involves cutaneous inflammation and keratinocytes hyperproliferation induced by an inflammatory cascade in dermis involving innate and adaptive immune cells. White adipose tissue located beneath the skin may contribute to the cutaneous inflammation by secreting adipokines and cytokines. The abnormal cutaneous and systemic expression of adipokines and cytokines could influence the activation, proliferation and differentiation of keratinocytes as well as immune cells contributing to the development of psoriatic lesions [4, 5]. Although the majority of inflammatory makers produced by adipose tissue and keratinocytes remains in the tissue, a small proportion of these biomarkers could also be identified in the systemic circulation [6]. These biomarkers measured in blood could be used to evaluate the disease severity and to monitor response to the treatment [7]. Recently, several studies have investigated the correlation of serum levels of adipokines and cytokines with psoriasis development and severity, but the results are controversial [8–12]. Moreover, some of these studies did not explore the effect of potential confounding factors, e.g. age, gender, ethnicity, on the association of adipokines and cytokines with psoriasis.

Therefore, we perform a meta-analysis of all eligible studies to compare the serum levels of 23 markers: tumour necrosis factor (TNF)-α, interferon (IFN)-γ, interleukin (IL)-1β, IL-2, IL-4, IL-6, IL-8, IL-10, IL-12, IL-17, IL-18, IL-21, IL-22, IL-23, adiponectin, chemerin, omentin, visfatin, lipocalin-2, resistin, soluble E-selectin (sE-selectin), fibrinogen, and complement 3 (C3), between psoriasis patients and healthy controls. Furthermore, we evaluate whether the strength of the association of these serum markers and risk of psoriasis varies by age, gender, ethnicity, psoriasis area and severity index (PASI), or psoriasis type.

## RESULTS

**Figure 1:**
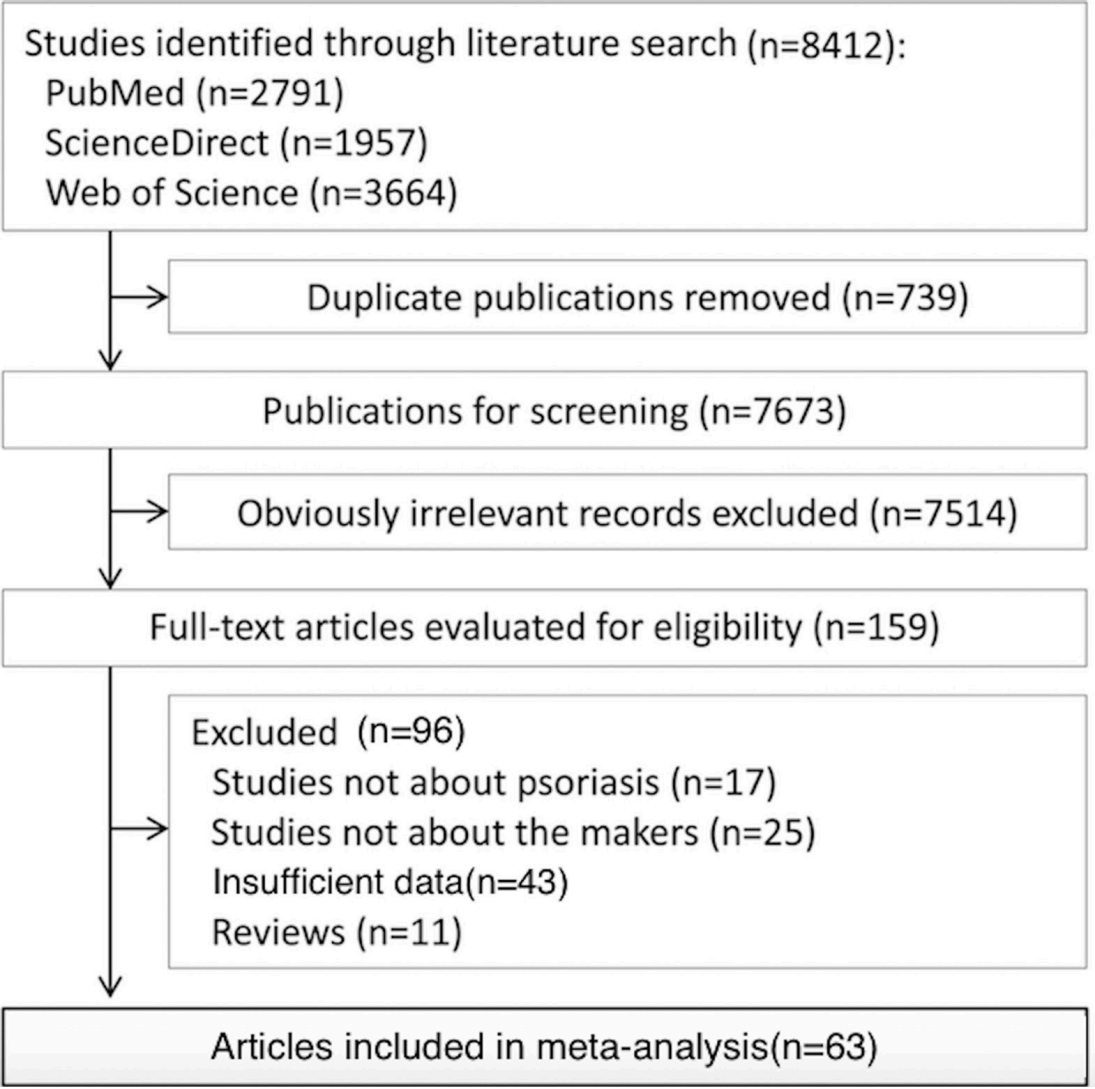
Flowchart for the selection of eligible studies

In total, 8412 publications matching the search criteria were identified. After the removal of duplicates, titles and abstracts of 7663 publications were screened, and 159 publications were determined to be potentially eligible. Based on the inclusion and excluding criteria, 63 articles were included in this meta-analysis (Figure 1) [8–70]. The characteristics of the included studies are shown in Supplementary Table 2.

Our study included 2876 psoriasis patients and 2237 healthy subjects. Among 63 studies, fifty-nine studies were case-control studies and four were cross-sectional studies, reporting the relationship between serum biomarker levels and psoriasis. Fifty-four studies were conducted in Caucasian populations and nine in Asian populations. The average age ranged from 23.0 to 60.8 in patient groups and 65.1 to 77.0 in control groups. A Psoriasis Area and Severity Index, PASI, was reported in 68.5% studies, with overall mean PASI 12.5 ± 7.8, and the average score from 5.2 to 26.7. Enzyme linked immunosorbent assay (ELISA) results were reported in 42 studies.

### Serum profile of pro-inflammatory cytokines in psoriasis patients

**Figure 2:**
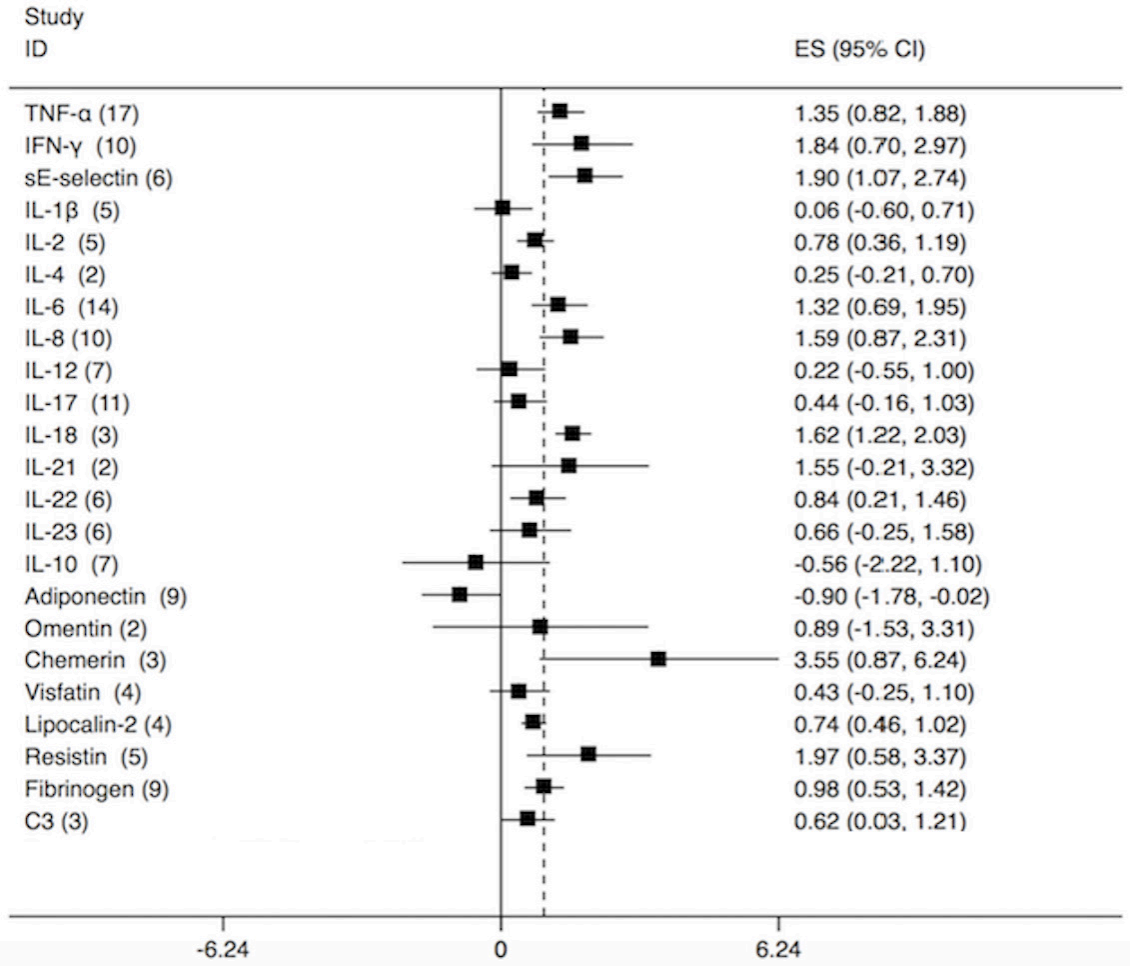
A forest plot of pooled standardized mean differences (SMDs) in serum levels of adipokines and cytokines The diamond represents the exact estimate from the study. The width of the line extending from each diamond represents the 95% confidence interval (CI). All individual study data and forest plots for all individual cytokines and adipokines are detailed in the Supplementary Files.

**Figure 3:**
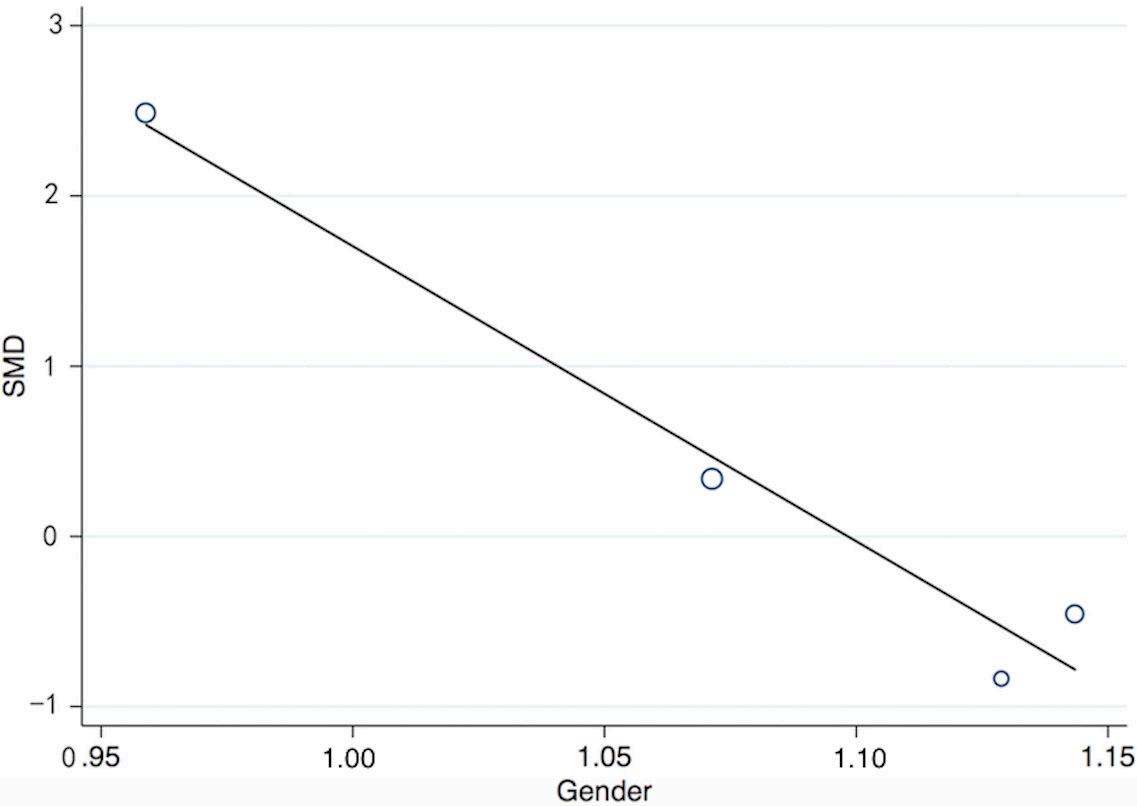
The meta-regression analysis for gender to assess differences in IL-17 serum levels between psoriasis patients and healthy controls

The serum levels of thirteen pro-inflammatory cytokines: TNF-α, IFN-γ, IL-1β, IL-2, IL-4, IL-6, IL-8, IL-12, IL-17, IL-18, IL-21, IL-22, and IL-23, were evaluated in this meta-analysis. The mean serum levels of seven of them: TNF-α, IFN-γ, IL-2, IL-6, IL-8, IL-18, and IL-22, were significantly higher in psoriasis patients than those in controls (Figure 2, Supplementary Table 3, Supplementary Figures 1–7; TNF-α: SMD = 1.35, 95% CI 0.82 to 1.88; IFN-γ: SMD = 1.84, 95% CI 0.70 to 2.97; IL-2: SMD = 0.77, 95% CI 0.36 to 1.19; IL-6: SMD = 1.32, 95% CI 0.69 to 1.95; IL-8: SMD = 1.59, 95% CI 0.87 to 2.31; IL-18: SMD = 1.62, 95% CI 1.22 to 2.03; IL-22: SMD = 0.84, 95% CI 0.21 to 1.46). The serum levels of remaining six inflammatory cytokines (IL-1β, IL-4, IL-12, IL-17, IL-21, and IL-23) did not show a significant difference between the psoriasis patients and control groups (Figure 2, Supplementary Figures 8–13; IL-1β: SMD = 0.06, 95% CI -0.59 to 0.71; IL-4: SMD = 0.25, 95% CI -0.21 to 0.70; IL-12: SMD = 0.22, 95% CI -0.55 to 1.00; IL-17: SMD = 0.43, 95% CI -0.16 to 1.03; IL-21: SMD = 1.55, 95% CI -0.21 to 3.31; IL-23: SMD = 0.66, 95% CI -0.25 to 1.58). Meta-regression involving adjustment for gender observed that the higher the percentage of men in the study, the larger the difference in IL-17 between psoriasis patients and healthy controls Figure 3, Supplementary Table 3; slope: -17.34, 95% CI -26.60 to -8.07; *P* = 0.02). Moreover, after adjustment for ethnicity, higher serum levels of IL-17 were associated with psoriasis in Asians, but not in Caucasians (Supplementary Table 1, Asians: SMD = 0.54, 95% CI -0.44 to 1.51, I^2^ = 96.1%; Caucasians: SMD = 0.27, 95% CI 0.04 to 0.49, I^2^ = 0%). No difference between Asians and Caucasians was observed for the correlation of other inflammatory cytokines with psoriasis. The increased serum levels of IL-2 were associated with other types of psoriasis, but not with psoriasis vulgaris (Supplementary Table 1, Others: SMD = 0.89, 95% CI 0.24 to 1.52, I^2^ = 81.9%; Psoriasis vulgaris: SMD = 0.57, 95% CI 0.16 to 0.98, I^2^ = 0%). However due to the low number of included studies, such correlation needs to be further investigated. Adjustments for age and study quality score did not alter the results (Supplementary Table 3).

### Serum profile of anti-inflammatory cytokines in psoriasis patients

One anti-inflammatory serum biomarker IL-10 was assessed in our meta-analysis. We found no significant difference of IL-10 serum concentrations between the psoriatic patients and healthy controls (Figure 2; Supplementary Figure 14; SMD = -0.56, 95% CI -2.22 to 1.10) [123]. The meta-regression by age, gender, PASI or study quality score and the stratified analysis across ethnicity, study design and psoriasis type showed that these factors did not significantly affect the results (Supplementary Table 3).

### Serum profile of other soluble factors

We also evaluated the relation of sE-selectin, fibrinogen, and C3 serum levels to psoriasis and found they were increased in psoriasis patients (Figure 2, Supplementary Figures 15–17, sE-selectin: SMD = 1.90, 95% CI 1.07 to 2.74; Fibrinogen: SMD = 0.98, 95% CI 0.53 to 1.42; C3: SMD = 0.62, 95% CI 0.03 to 1.21). The results of meta-regression for gender showed a borderline association of the higher fibrinogen serum level with the higher percentage of men in the study (Supplementary Table 3, slope: -4.01, 95% CI -8.34 to 0.31; *P* = 0.06). The results of subgroup analysis by age, ethnicity, study design, and psoriasis type did not show the significant differences (Supplementary Table 3, Supplementary Table 1).

### Serum profile of adipokines

Subsequently, we evaluated the association between serum levels of adipokines (adiponectin, omentin, chemerin, visfatin, lipocalin-2, and resistin) and psoriasis. The mean value of adiponectin serum levels was significantly lower, whereas the mean values of lipocalin-2, chemerin and resistin serum levels were significantly higher in psoriasis patients than that in healthy controls (Figure 2; Supplementary Figures 18–21, Adiponectin: SMD = -0.90, 95% CI -1.78 to -0.02; Lipocalin-2: SMD = 0.74, 95% CI 0.46 to 1.02; Chemerin: SMD = 3.55, 95% CI 0.86 to 6.24; Resistin: SMD = 1.97, 95% CI 0.58 to 3.37). No significant differences in serum levels of visfatin and omentin were observed between the psoriasis patients and healthy controls (Figure 2, Supplementary Figure 22, Supplementary Figure 23, Visfatin: SMD = 0.43, 95% CI -0.25 to 1.10; Omentin: SMD = 0.89, 95% CI -1.53 to 3.31;). The results of subgroup analysis by ethnicity revealed that the lower serum levels of adiponectin were observed in Caucasians, than in Asians (Supplementary Table 1, Caucasians: SMD = -1.17, 95% CI -2.22 to -0.12; I^2^ = 97.30%; Asians: SMD = 0.03, 95% CI -1.64 to 1.69; I^2^ = 94.10%). Moreover, no association with psoriasis was found in serum levels of lipocalin-2 in Caucasians (Supplementary Table 1, Caucasians: SMD = 0.85, 95% CI 0.58 to 1.12, I^2^ = 0). Subgroup analysis by psoriasis type found significant differences in adiponectin serum levels between the psoriasis patients and healthy controls (Supplementary Table 1, Psoriasis vulgaris: SMD = -1.29, 95% CI -2.55 to -0.03; I^2^ = 97.7%, Others: SMD = -0.15, 95% CI -1.14 to 0.84, I^2^ = 90.7%). The subgroup and meta-regression analyses found that age, gender, PASI, and study quality score did not significantly affect the results (Supplementary Table 3).

### Sensitivity analysis and publication bias

The sensitivity analysis revealed that the results remained stable and reliable. The Begg’s funnel plot and Egger’s test found no evidence of publication bias (Supplementary Table 3).

## DISCUSSION

Our meta-analysis showed systemic inflammation in psoriasis patients compared with healthy individuals, with 13 of 23 analysed pro-inflammatory soluble factors (TNF-α, IFN-γ, sE-selectin, IL-2, IL-6, IL-8, IL-18, IL-22, chemerin, lipocalin-2, resistin, fibrinogen and C3) being increased and one anti-inflammatory marker (adiponectin) being decreased in psoriasis patients. Serum levels of IL-1β, IL-4, IL-10, IL-12, IL-17, IL-21, IL-23, visfatin and omentin were not significantly different in patients and controls. The meta-regression by gender observed higher serum levels of IL-17 between psoriasis patients and controls. For other biomarkers, age, gender, study quality and PASI do not explain the differences in effect size between the studies.

Adipose tissue is located under the skin and contains adipocytes, a variety of immune cells and other cells including the vascular endothelial cells. In recent years, adipose tissue has been recognized as a major endocrine organ producing bioactive mediators, such as adipokines (including adiponectin or resistin), chemokine and cytokines (including TNF-α, IL-6 and IL-8). Given adipose tissue properties and its widespread localization, it was suggested to affect skin immune system and contribute to the pathogenesis of psoriasis [20, 71, 72, 75]. In the current meta analysis we compared the levels of adipokines (adiponectin, omentin, chemerin, visfatin, lipocalin-2 and resistin), circulating cytokines (TNF-α, IFN-γ, IL-1β, IL-2, IL-4, IL-6, IL-8, IL-10, IL-12, IL-17, IL-18, IL-21, IL-22 and IL-23) and other inflammatory molecules (sE-selectin, fibrinogen, C3) between psoriasis patients and healthy individuals.

The adipokines can play anti-inflammatory or pro-inflammatory roles in the systemic inflammation in psoriasis patients [74]. In inflammatory state underlying psoriasis, resistin produced mainly by monocytes and macrophages in adipose tissue, could stimulate the secretion of pro-inflammatory cytokines, TNF-α and IL-12, by monocytes in NF-kB-dependent manner. Resistin increases expression of adhesion molecules and production of chemokines in endothelial cells contributing to immune cells infiltration [87, 88]. The expression of resistin is modulated by IL-1β and IL-6 [29, 73]. Chemerin, mainly produced by white adipose tissue, is also expressed by keratinocytes and endothelial cells [89–91]. Its production is increased by IL1β and TNF-α [92–94]. Chemerin stimulates adipocytes and endothelial cells and may increase angiogenesis in psoriatic skin [90, 95, 96]. Chemerin can induce chemotaxis of plasmatoid dendritic cells (pDCs) and immature myeloid DCs in developing skin lesions of psoriasis patients [82].

Lipocalin-2, known as neutrophil gelatinase-associated lipocalin, is a protein stored in the specific granules of human neutrophils [97]. It is regarded as an antimicrobial protein against bacterial infection by sequestrating iron [98]. The other well-established biologic functions of Lipocalin-2 include regulating diverse cellular processes, such as cell growth and migration/invasion [99–101]. In addition, Lipocalin-2 plays a role in the inflammation enhancing the production of pro-inflammatory cytokines including IL-6 and IL-8 [102]. Its expression can be induced by IL-17 [103]. Lipocalin-2 was expressed at a higher level in psoriatic skin that induced the secretion of pro-inflammatory factors and chemotaxis of neutrophils [104, 105]. However, its exact effect in the pathophysiology of psoriasis requires further study [36]. Visfatin is produced by adipose tissue, macrophages, dendritic cells and epithelial cells. It was shown to induce IL-1β, TNF-α, and IL-6 production by monocytes [106]. Visfatin can promote the expression of a variety of inflammatory cytokines by keratinocytes in NF-κB-dependent manner. Moreover, it can induce endothelial proliferation by upregulating the secretion of vascular endothelial growth factor in endothelial cells [83].

Adiponectin is produced by white adipose tissue, mainly by adipocytes [107–109]. It acts on various cell types including adipocytes, keratinocytes, monocytes, T lymphocytes, and endothelial cells. In general, adiponectin plays anti-inflammatory and protective roles. It was shown to inhibit production of pro-inflammatory cytokines like TNF-α, IL-6, IL17 and to increase the production of anti-inflammatory factors including IL-10 [110, 111]. In addition, adiponetin can suppress the vascular inflammation by inhibiting the expression of endothelial cell adhesion molecules (e.g. E-selectin) [72, 73, 81]. Omentin is produced in adipose tissue and its main producers are assumed to be endothelial cells [112, 113]. It acts on adipocytes and endothelial cells. Similar to adiponectin, omentin plays anti-inflammatory role by suppressing TNF-α-stimulated expression of pro-inflammatory factors in vascular endothelial cells [114]. Moreover, blood levels of omentin and adiponectin decreased in obesity [74].

We found that serum levels of three proinflamatory adipokines (chemerin, lipocalin-2 and resistin) were increased, while serum levels of one anti-inflammatory adipokine, adiponectin, were decreased in psoriasis patients. Contrary to what was expected, pooled serum levels of anti-inflammatory omentin were not significantly decreased in psoriasis patients. This could be due to only two included studies that in addition found opposite effects in the association of omentin levels with psoriasis. Omentin and adiponectin share anti-inflammatory properties. Moreover, plasma levels of omentin and adiponectin are positively correlated [115, 116]. More studies are needed to evaluate the association of blood levels of omentin with psoriasis.

Resistin and adiponectin could be potential biomarkers for the response to the anti-psoriatic treatment. Resistin was significantly increased in psoriasis patients (Figure 2, Supplementary Figure 22) and its plasma levels decreased upon anti-psoriatic treatment [35, 117, 118]. Adiponectin was significantly decreased in psoriasis patients (Figure 2, Supplementary Figure 18) and its plasma levels increased in response to anti-psoriatic treatment [119–122]. Previous studies have found that serum adipokine levels were positively related to PASI score, however our meta-analysis does not support it [31, 35]. In addition, our study showed that the differences were overall independent of age and gender.

**Figure 4:**
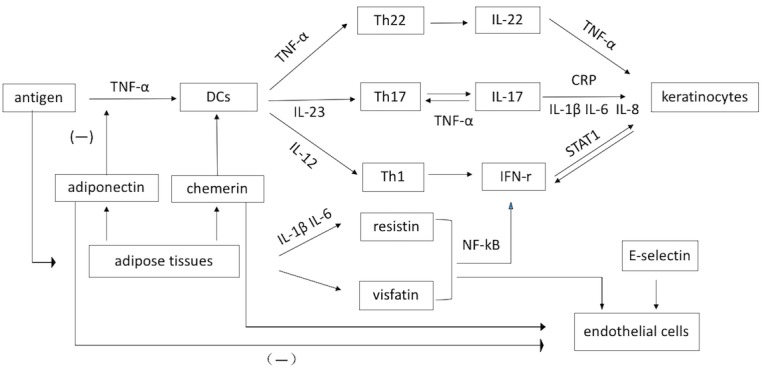
A schematic representation of role of the inflammatory factors in psoriasis

In psoriasis, there is a cutaneous and systemic overexpression of various inflammatory cytokines and these cytokines could impact each other [4, 8, 9]. Once the cutaneous inflammation is stimulated by the antigen, macrophages, keratinocytes, Th1 cells, T17 cells, Th22 cells and BDCA-1−inflammatory DCs will produce TNF-α, which plays an important role in the inflammatory process in psoriasis (Figure 4) [84]. TNF-α stimulates the migration of Langerhans cells by lowering the level of e-cadherin, and is involved in the NF-κB-mediated inflammation pathway, which contributes to cell survival, proliferation, and transcription of antiapoptotic factors [85]. Moreover, TNF-α can enhance the expression of IL-6, IL-8, C-reactive protein, which mediate T cell activation, the acute-phase inflammation reaction and provide a powerful signal for the concentration of neutrophils [86]. IFN-γ is mainly produced by Th1 cells and can activate the signal transducer and activator of transcription (STAT) 1, which regulates many genes expressed in psoriatic skin lesions [84]. In addition, the transcription of IFN-γ and TNF-α can be regulated by IL-2 and IL-12 [76]. IL-2 plays a role in the differentiation, proliferation, and maturation of T cells into effector and memory T cells. IL-18 plays an important role in cell adhesion and can stimulate the release of IFN-γ [86]. It is worth noting that IL-22 produced by Th17 and mainly Th22 cells has been observed to stimulate hyperplasia and abnormal differentiation of keratinocytes. In addition, TNF- α, IL-1β, IL-6, and INF-γ have been found to increase the production of C3 from the liver and probably from adipose tissue in psoriasis patients. Fibrinogen is a multifunctional circulating glycoprotein involved in inflammation [23, 68, 77, 78]. IL-17 expression is activated by IL-23 and may be inhibited by IL-12. IL-17 has been shown to induce TNF-α, IL-1β, IL-6, IL-10 and IL-12. IL-21, produced by activated CD4+ T cells and NKT cells, can promote cell proliferation and mediation of cellular and humoral immunities [11].

The present meta-analysis found that TNF-α, IFN-γ, sE-selectin, IL-2, IL-6, IL-8, IL-18, IL-22, fibrinogen and C3 were elevated in serum of patients with psoriasis, indicating that serum levels of these cytokines may correlate to their levels in tissue. Therefore, they could act as a novel biomarker to monitor disease occurence. In addition, our study found IL-17 as a risk factor for occurrence of psoriasis in men compared with women, but the mechanisms underlying the gender difference remains unclear.

### Study limitations

Some potential limitations could affect the interpretation of our findings. First, we did not include the studies using interquartile ranges and medians. The sample size in these studies was not large, so the distribution of the data might not be symmetrical. Thus, the median was not similar to the mean and it was not possible to estimate a standard deviation from an interquartile range. Although, it limited the sample size, the pooled results might be more precise. Second, there was a significant heterogeneity between the included studies, which could reduce the reliability of our results. Therefore, stratified, sensitivity and meta-regression analyses were carried out to explore potential source of heterogeneity, which could strengthen our analysis. Third, the analysis was primarily based on unadjusted data and did not control for confounding factors including age, gender, and BMI, and therefore cannot exclude the influence of mixed factors. Several inflammatory adipokines and cytokines have been demonstrated to be associated with the pathogenesis of psoriasis in relation to BMI, which exerted a key role in development of psoriasis. We have performed meta-regression analyses adjusted for age and gender, but not for BMI due to insufficient amount of available data. Finally, although apparent publication bias was not found using statistical tests, the potential publication bias could be a concern. It is challenging to completely rule out publication bias, and more studies are needed for its careful evaluation.

In conclusion, serum levels of TNF-α, IFN-γ, IL-2, IL-6, IL-8, IL-18, IL-22, chemerin, lipocalin-2, resistin, sE-selectin, fibrinogen and C3 were significantly elevated and serum level of adiponectin was significantly decreased in psoriasis patients compared to healthy individuals. Our findings suggested that the difference was overall independent of age, gender, study quality and PASI, except gender difference observed in serum levels of IL-17. The analyzed cytokines and adipokines can be used as markers for disease detection and to evaluate the efficacy of anti-psoriatic treatment. As serum levels of most of the analyzed factors are largely independent of psoriasis type and severity, they are unsuitable as markers to monitor disease progression. Future, carefully designed studies with large study populations are warranted to further evaluate the association of adipokines and cytokines serum levels with the pathogenesis of psoriasis.

## MATERIALS AND METHODS

### Study identification

This review was registered in PROSPERO (registration number: CRD 42017057297). Our meta-analysis was in accordance with the criteria for systematic reviews and meta-analyses [40].

A computer-based literature search was performed to identify the available relevant studies published before December 1, 2016 in PubMed, ScienceDirect and Web of Science, using the terms “IFN-γ or TNF-α or sE-selectin or IL-1β or IL-2 or IL-4 or IL-6 or IL-8 or IL-10 or IL-12 or IL-17 or IL-18 or IL-21 or IL-22 or IL-23 or adiponectin or omentin or chemerin or visfatin or resistin or fibrinogen or lipocalin-2 or C3” and “serum” and “psoriasis”. The publication language was limited to English. The reference lists of retrieved articles were hand searched to find additional relevant studies.

### Study selection

We determined inclusion and exclusion criteria before the search. The inclusion criteria were as follows: study design was limited to cross-sectional study, case-control study and cohort study; patients had to meet the diagnostic criteria for psoriasis; and the study provided the results as mean and standard deviation (SD) or original data to calculate mean and SD in a patient group and a control group. The exclusion criteria were as follows: the exposure included psoriatic arthritis; the study provided only the plasma biomarker concentration; the studies showed only median and interquartile range; and the psoriasis patients were under 18 years of age. If multiple publications reported results from the same study population, we included only the publication with the most subjects.

### Data abstraction and quality assessment

Two independent investigators (F.B. and W.Z.) abstracted data using a standard data extraction form, and any disagreement was resolved by a third author (H.M.). The following information was extracted from each included article: first author, year of publication, study location, study design, number of cases and controls, psoriasis area and severity index (PASI), the mean age, male versus female proportion, ethnicity, psoriasis type, and the relevant serum biomarker level (mean ± SD).

The study quality was assessed using the Newcastle–Ottawa Scale, categorized into three groups: the selection of the study groups (4 quality items), the comparability of the groups (2 items), and ascertainment of exposure (3 items). The scores ranged from 0 (the worst) to 9 (the best).

### Statistical analysis

The expected outcome from each study was the difference in mean levels of serum marker between psoriasis patients and healthy controls. Because of differences in the measurement methods and units, standardized mean difference (SMD) with 95% confidence intervals (CI) was calculated using Cohen’s d in random-effects or fixed effects models. The between-study heterogeneity was evaluated by Q and I2 statistics. A random-effects model was used when the significant heterogeneity with *P* < 0.05 or I^2^ > 50% existed; otherwise, a fixed-effects model was chosen. To explore potential sources of heterogeneity, subgroup analysis was performed according to race (Caucasians vs. Asians), study design (Case control vs. Cross-sectional), and type of psoriasis (Psoriasis vulgaris vs. Others). We also conducted a meta-regression for age, gender, PASI and study quality score. In addition, a sensitivity analysis was performed to assess the stability of the pooled results by removing one study at a time. Publication bias was evaluated using Egger’s regression test and Begg’s adjusted rank correlation test [79, 80]. All statistical analyses were carried out with Stata version 11.0 (STATA, College Station, TX, USA). *P* value of < 0.05 was considered statistically significant.

## SUPPLEMENTARY MATERIALS FIGURES AND TABLES








