# Supplementary material for: Serum levels of adipokines and cytokines in psoriasis patients: a systematic review and meta-analysis
Source: Oncotarget. 2017 Nov 1;9(1):1266–78. doi: 10.18632/oncotarget.22260 (PMC5787437; doi:10.18632/oncotarget.22260)
Supplement: Supplementary file 1 [file oncotarget-09-1266-s001.pdf]

Serum levels of adipokines and cytokines in psoriasis patients: a systematic review and meta-analysis

SUPPLEMENTARY MATERIALS

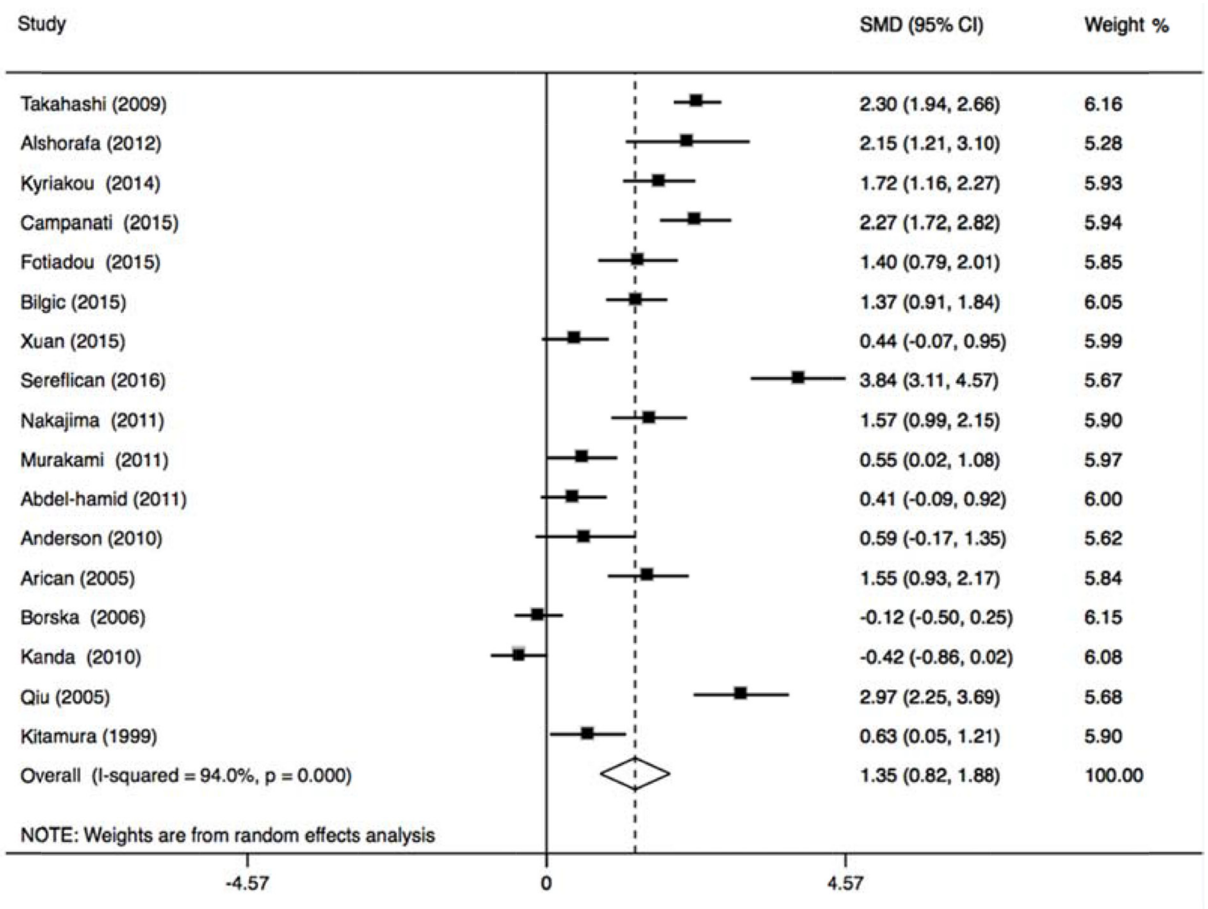

Supplementary Figure 1: Forest plot of the differences in serum levels of TNF-α between psoriasis patients and healthy controls.

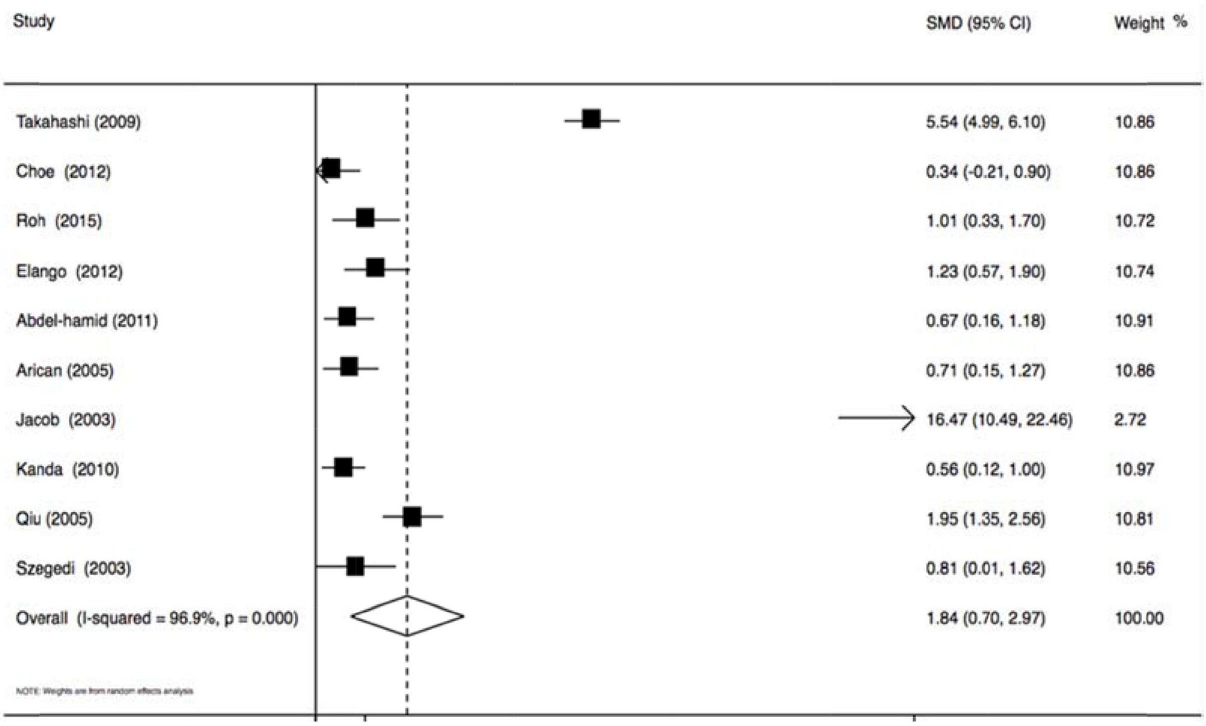

Supplementary Figure 2: Forest plot of the differences in serum levels of IFN-γ between psoriasis patients and healthy controls.

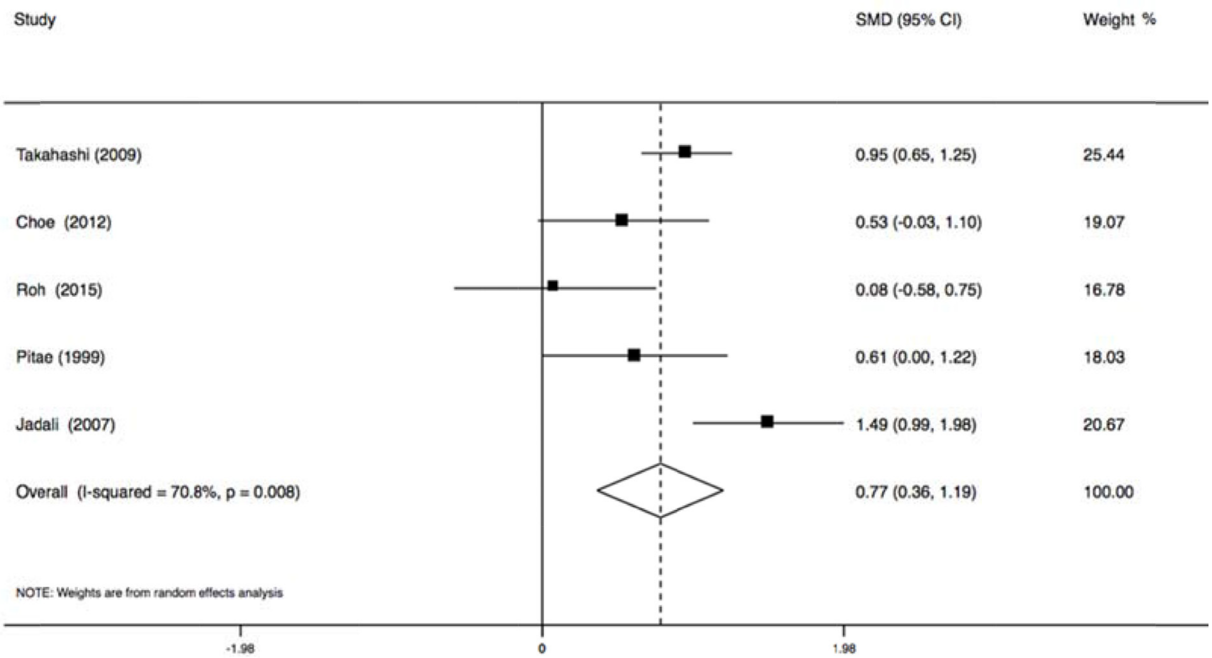

Supplementary Figure 3: Forest plot of the differences in serum levels of IL-2 between psoriasis patients and healthy controls.

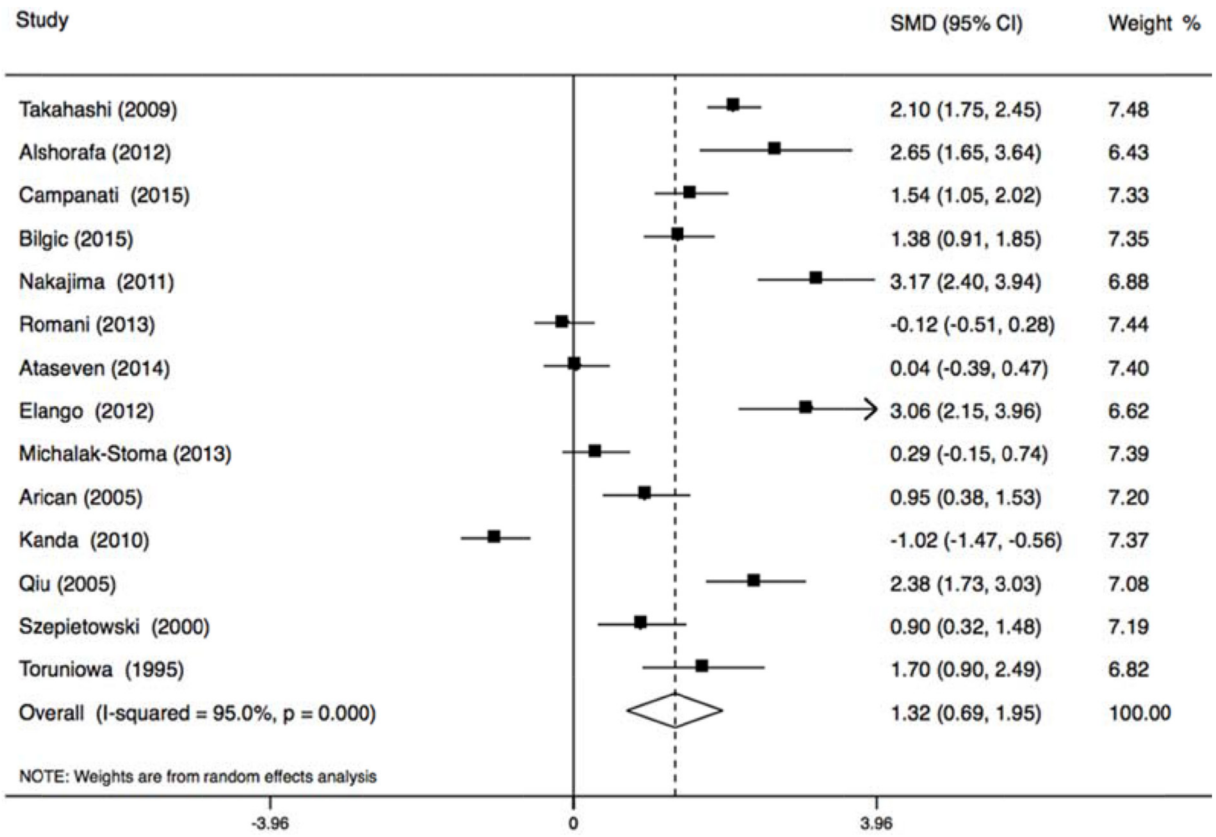

Supplementary Figure 4: Forest plot of the differences in serum levels of IL-6 between psoriasis patients and healthy controls.

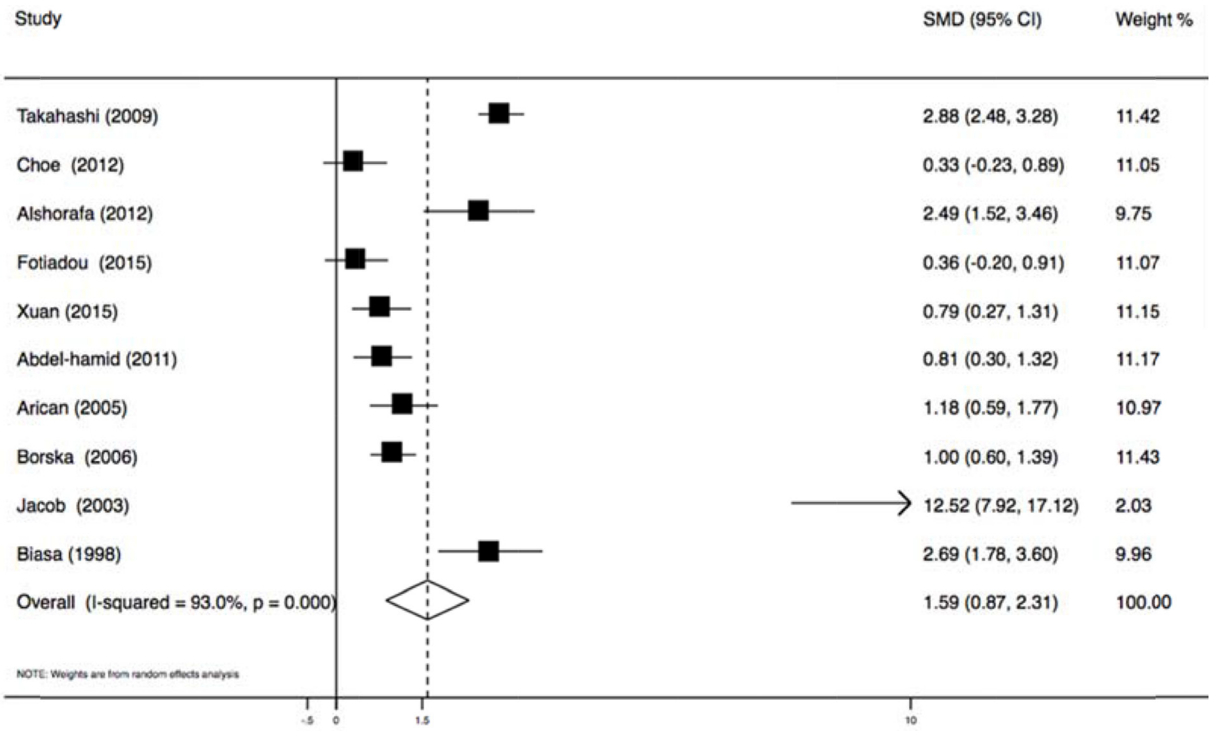

Supplementary Figure 5: Forest plot of the differences in serum levels of IL-8 between psoriasis patients and healthy controls.

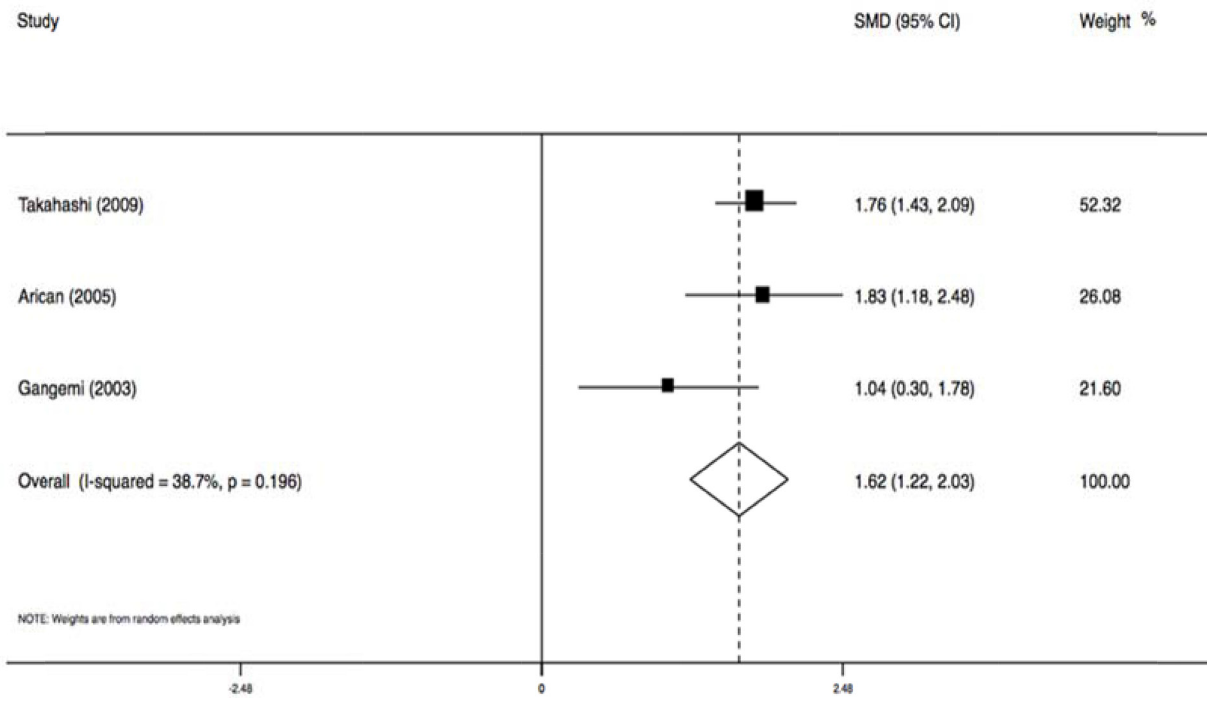

Supplementary Figure 6: Forest plot of the differences in serum levels of IL-18 between psoriasis patients and healthy controls.

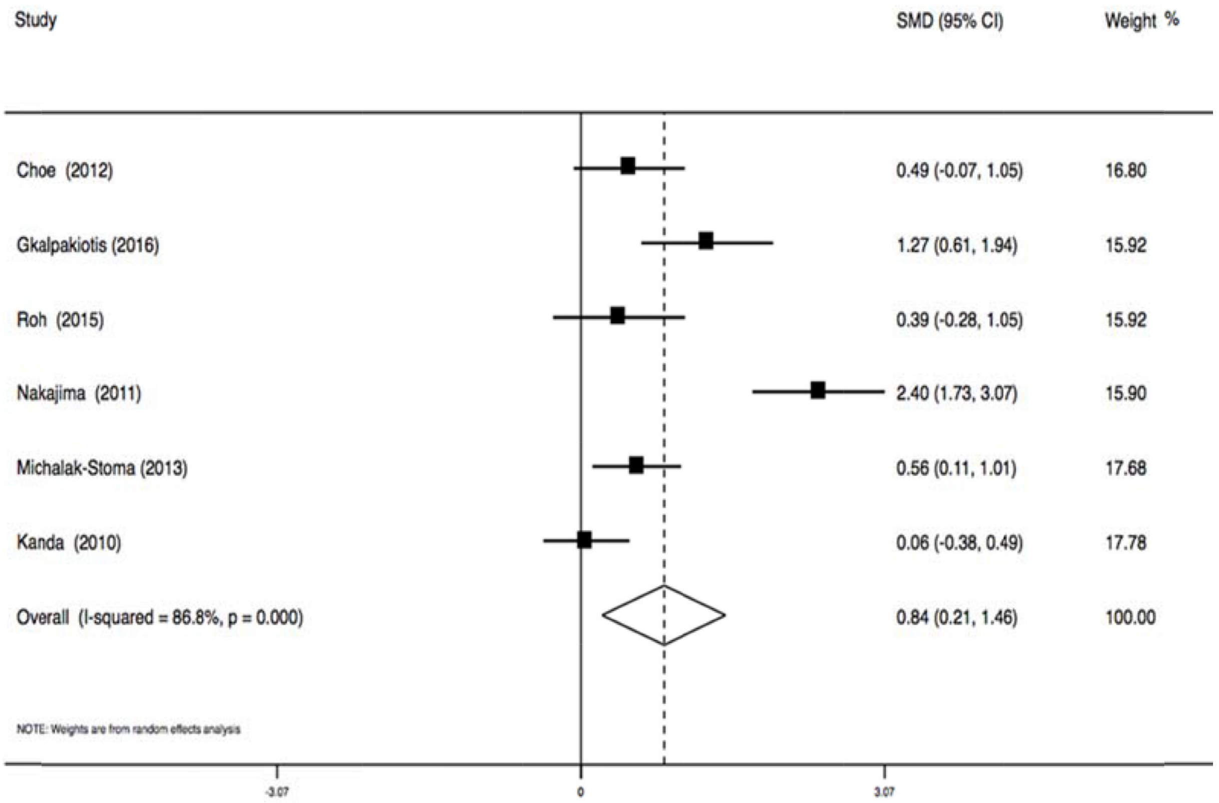

Supplementary Figure 7: Forest plot of the differences in serum levels of IL-22 between psoriasis patients and healthy controls.

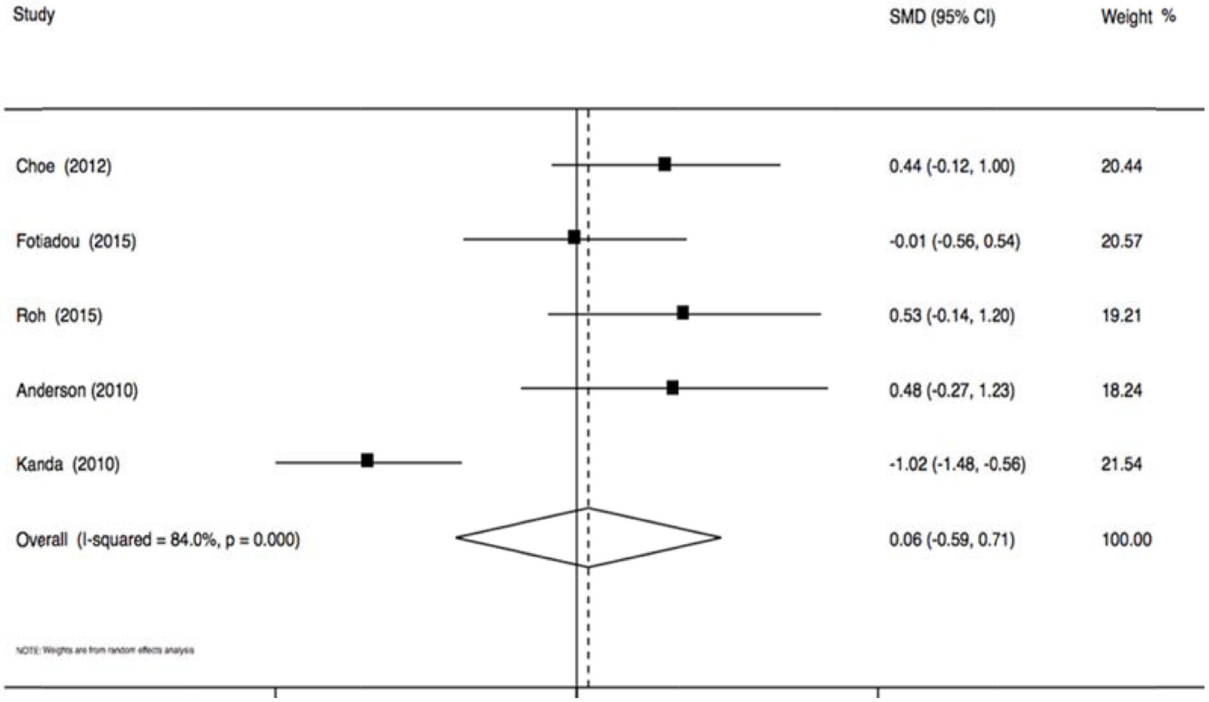

Supplementary Figure 8: Forest plot of the differences in serum levels of IL-1β between psoriasis patients and healthy controls.

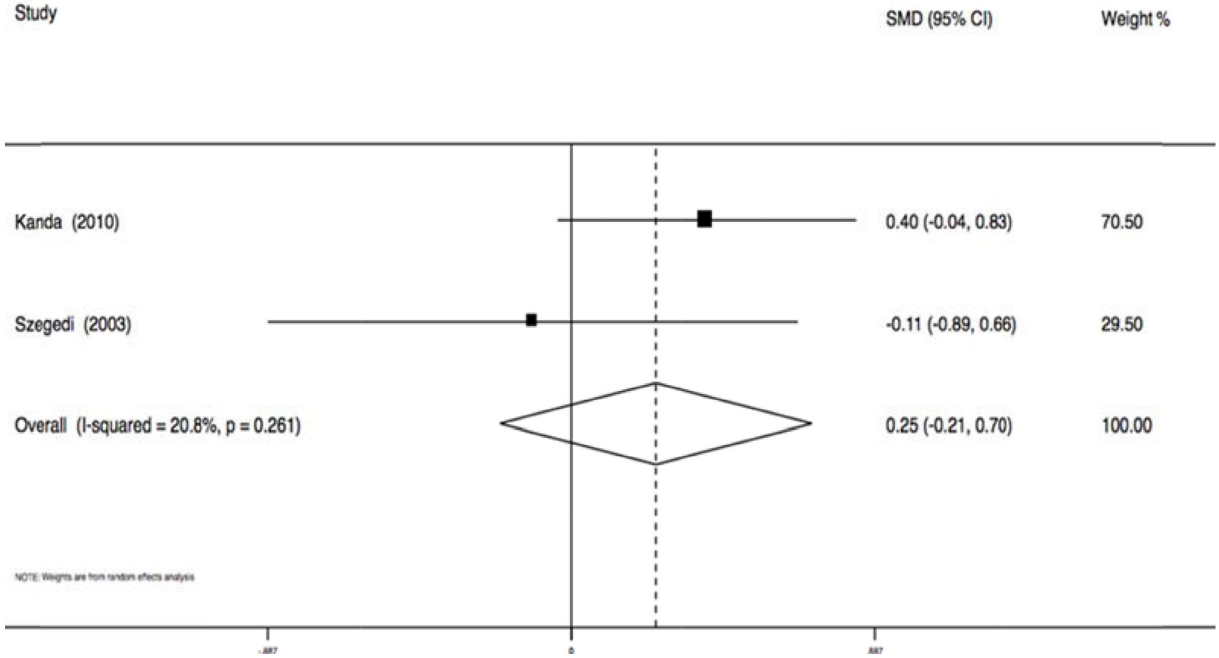

Supplementary Figure 9: Forest plot of the differences in serum levels of IL-4 between psoriasis patients and healthy controls.

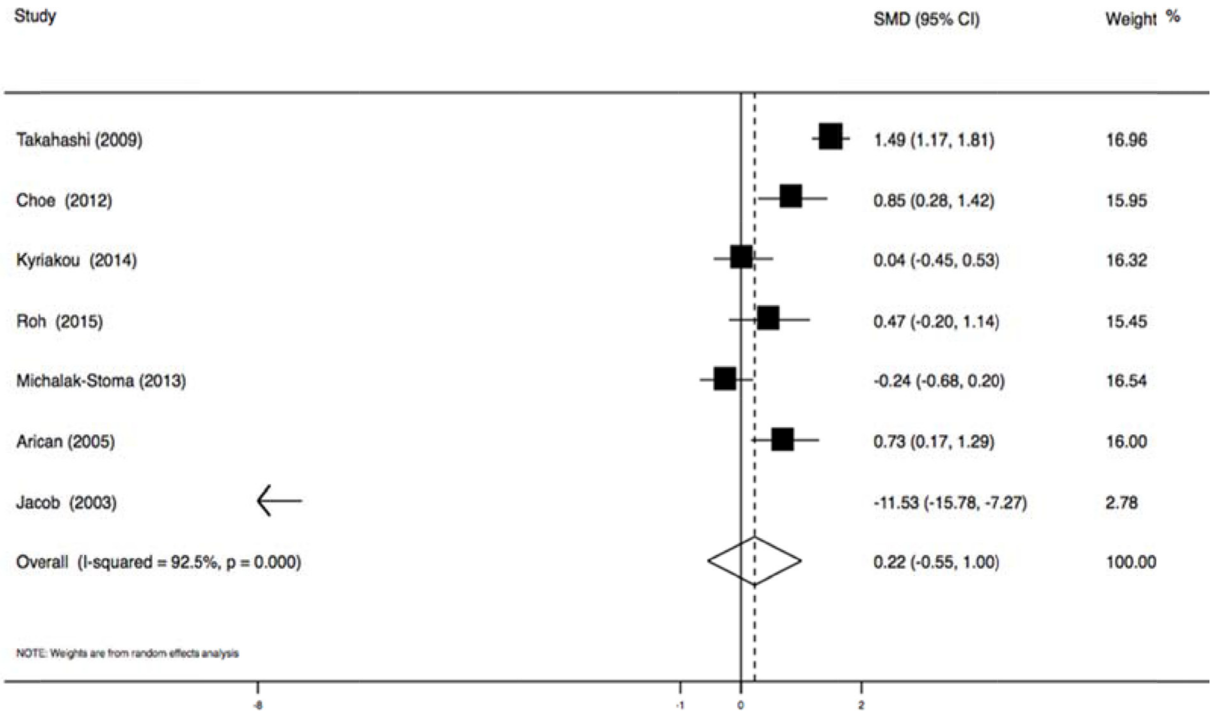

Supplementary Figure 10: Forest plot of the differences in serum levels of IL-12 between psoriasis patients and healthy controls.

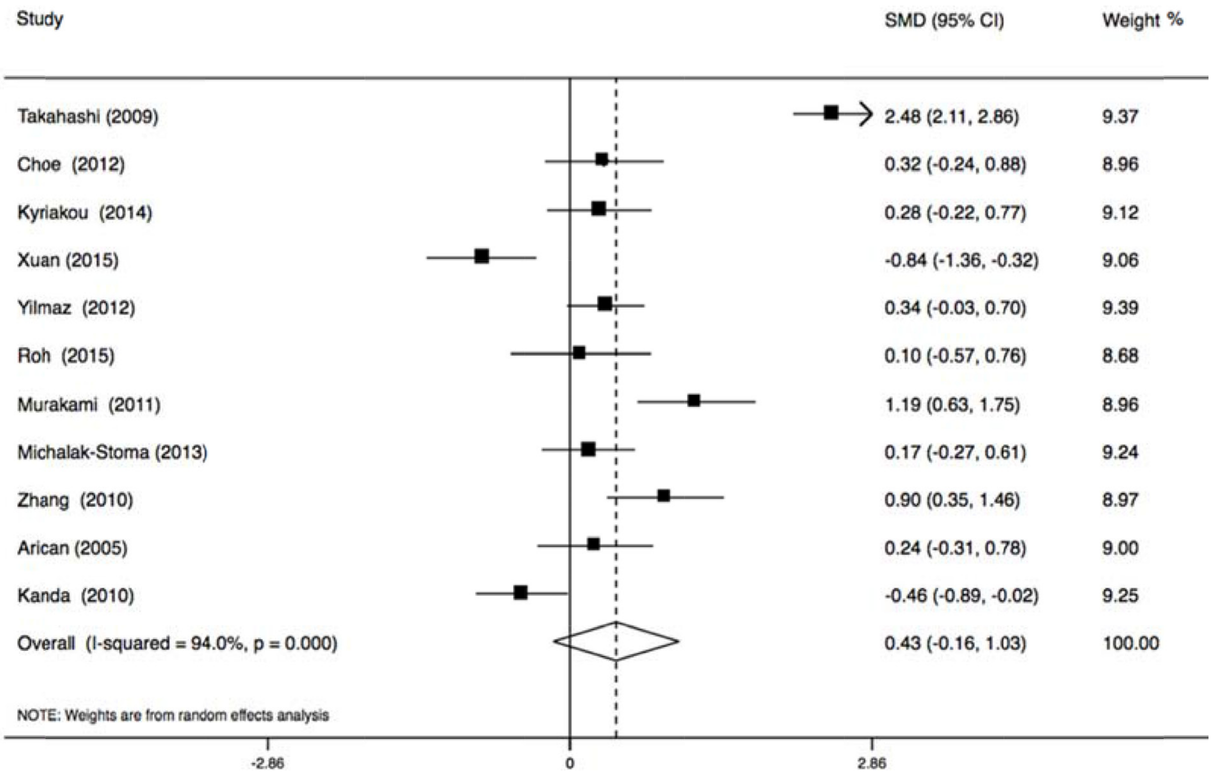

Supplementary Figure 11: Forest plot of the differences in serum levels of IL-17 between psoriasis patients and healthy controls.

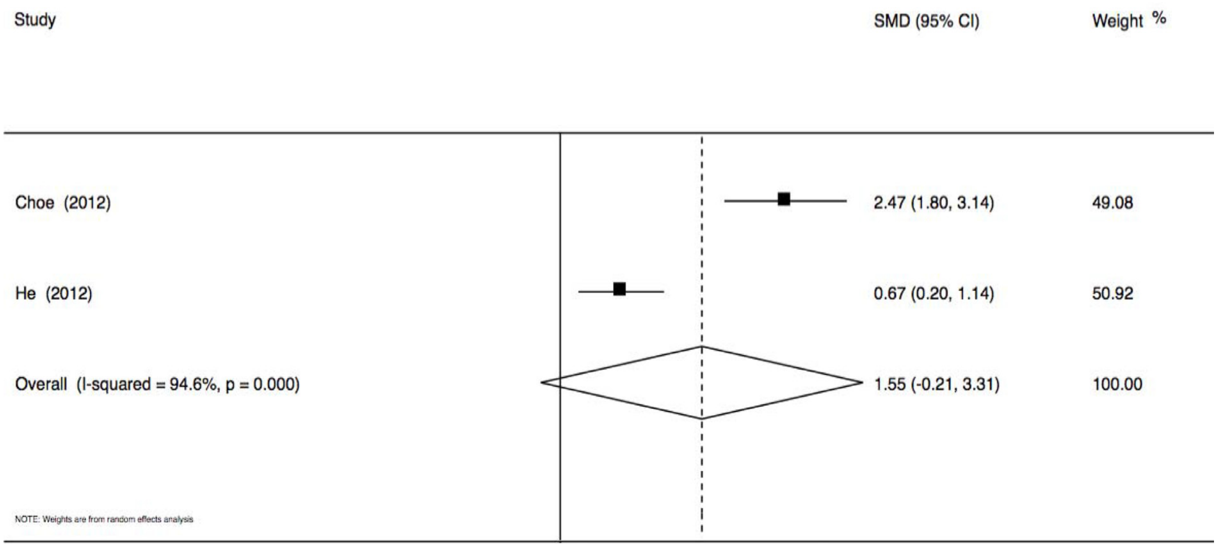

Supplementary Figure 12: Forest plot of the differences in serum levels of IL-21 between psoriasis patients and healthy controls.

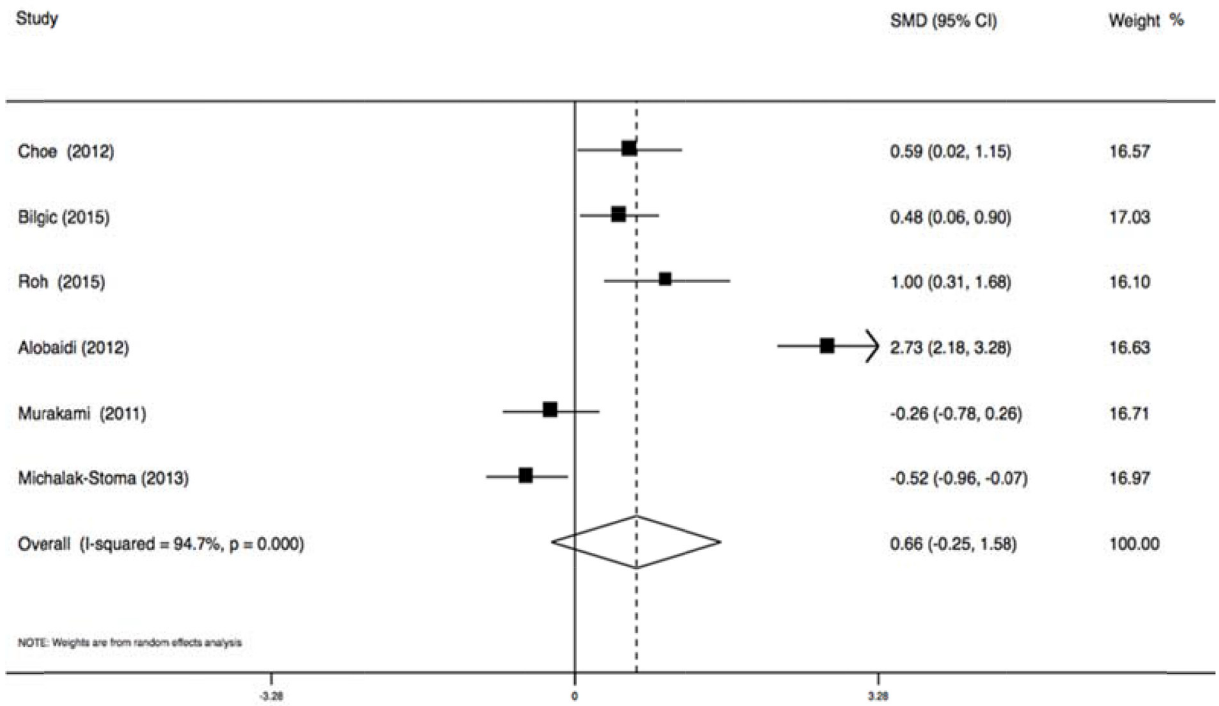

Supplementary Figure 13: Forest plot of the differences in serum levels of IL-23 between psoriasis patients and healthy controls.

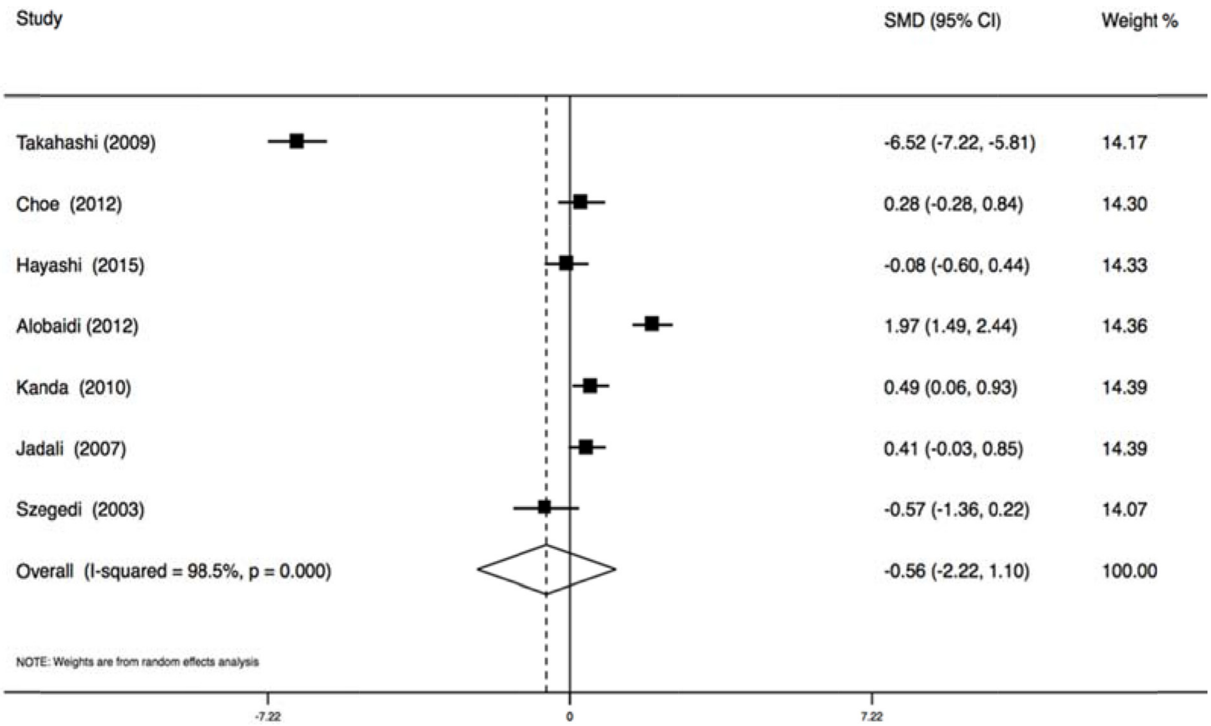

Supplementary Figure 14: Forest plot of the differences in serum levels of IL-10 between psoriasis patients and healthy controls.

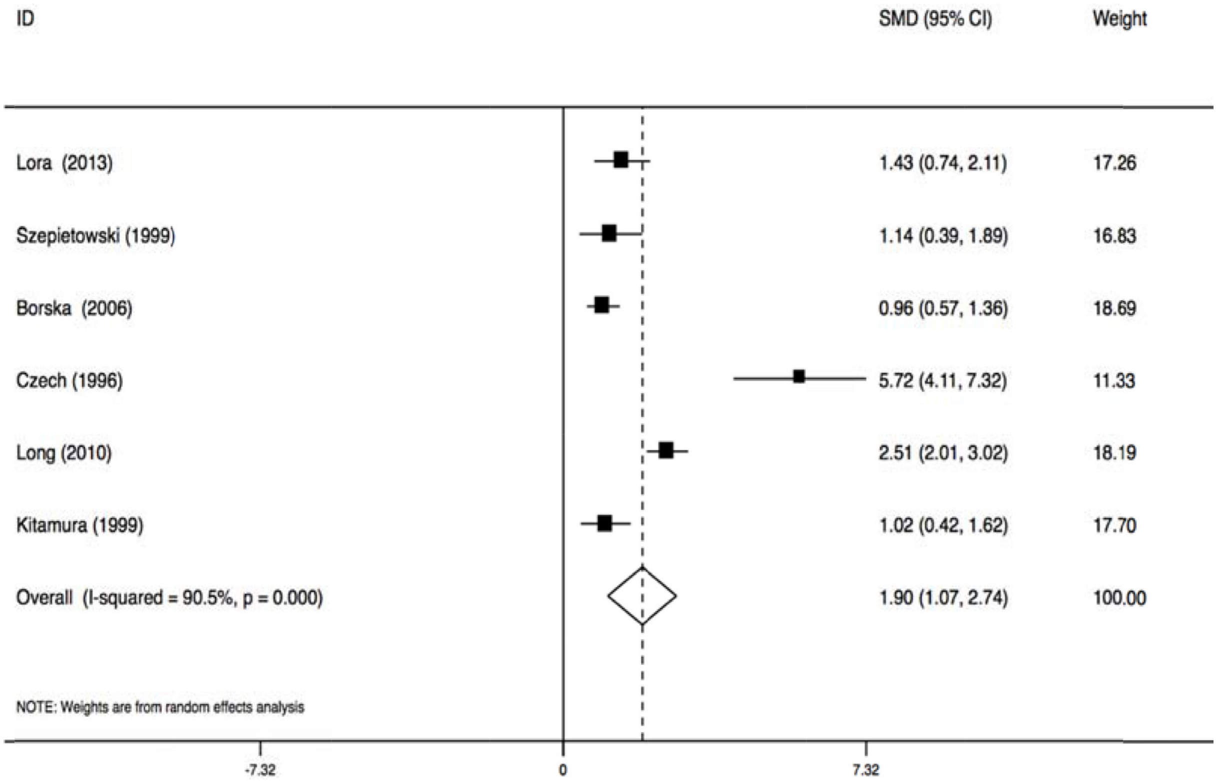

Supplementary Figure 15: Forest plot of the differences in serum levels of sE-selectin between psoriasis patients and healthy controls.

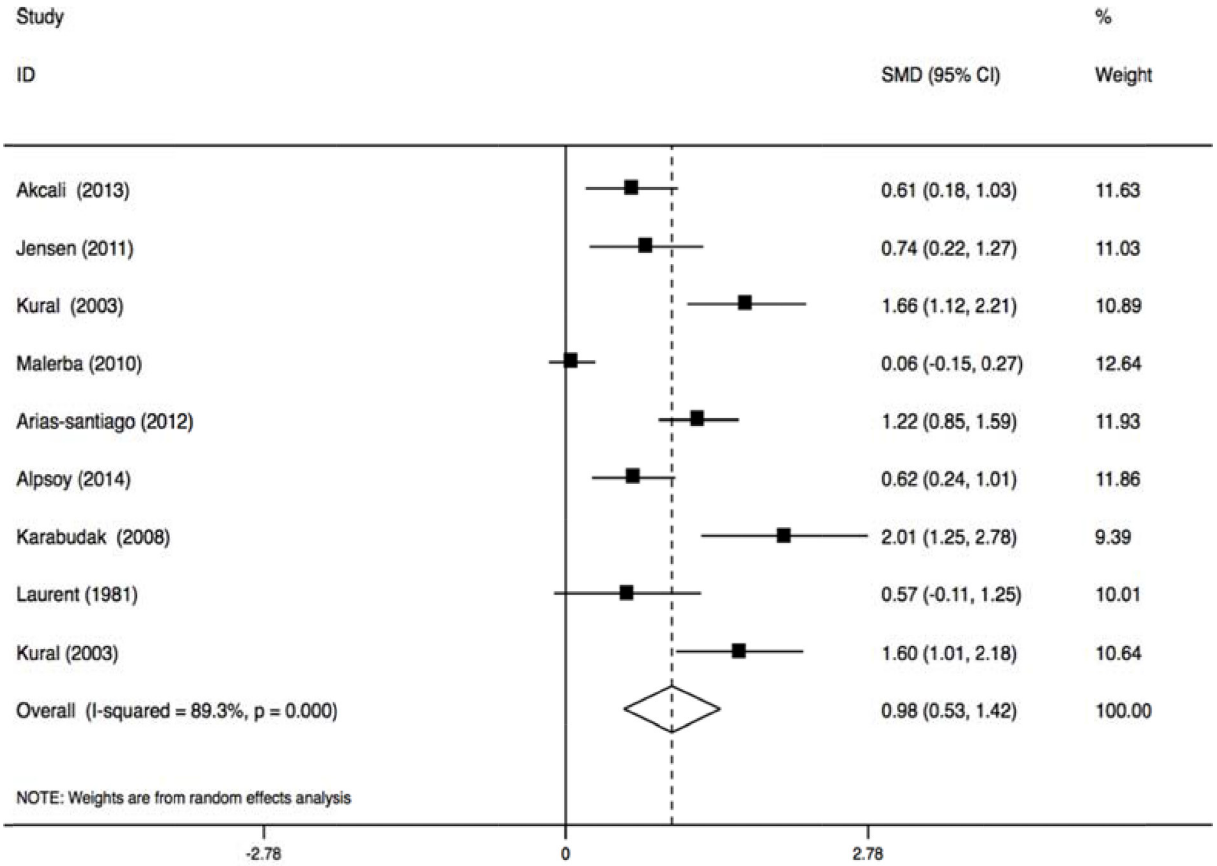

Supplementary Figure 16: Forest plot of the differences in serum levels of fibrinogen between psoriasis patients and healthy controls.

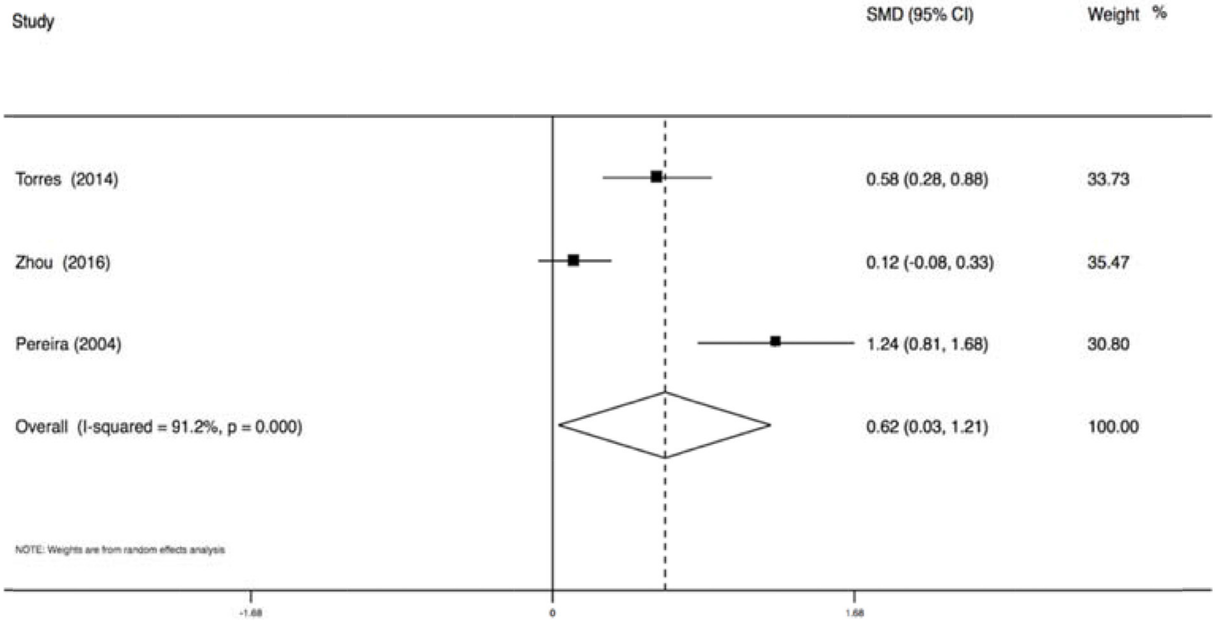

Supplementary Figure 17: Forest plot of the differences in serum levels of Complement 3 between psoriasis patients and healthy controls.

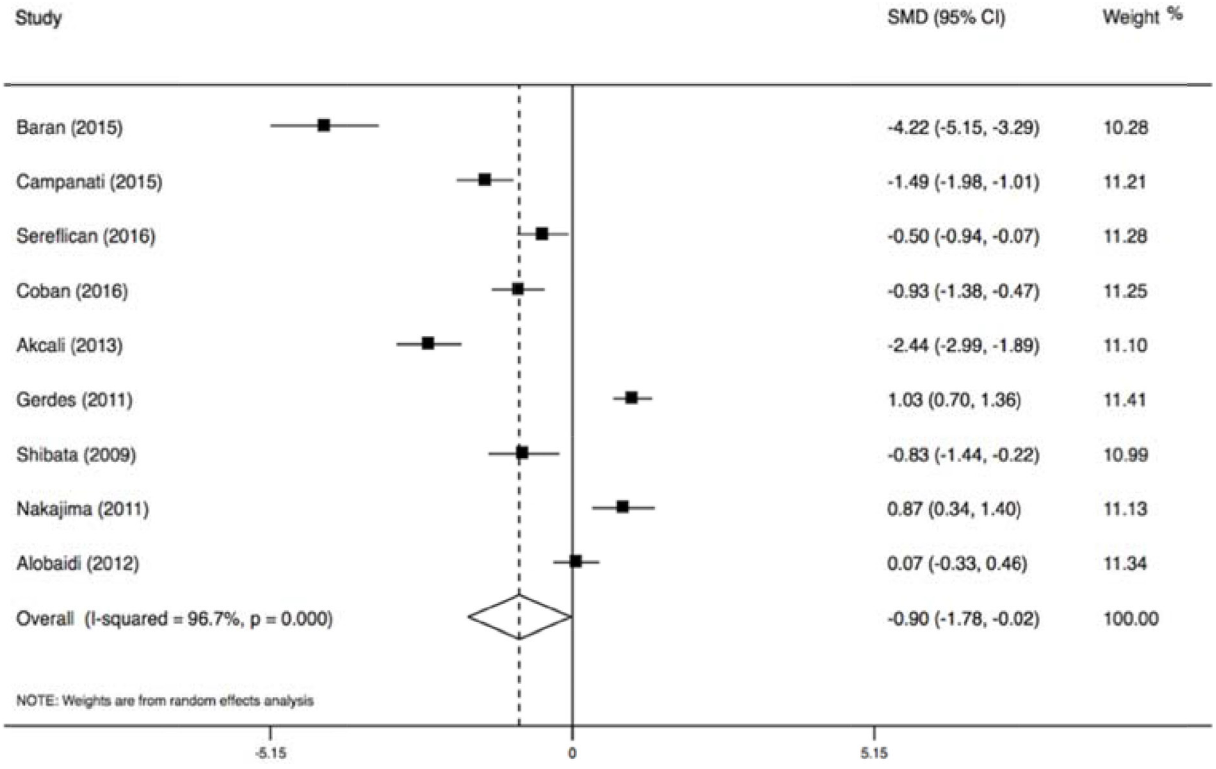

Supplementary Figure 18: Forest plot of the differences in serum levels of adiponectin between psoriasis patients and healthy controls.

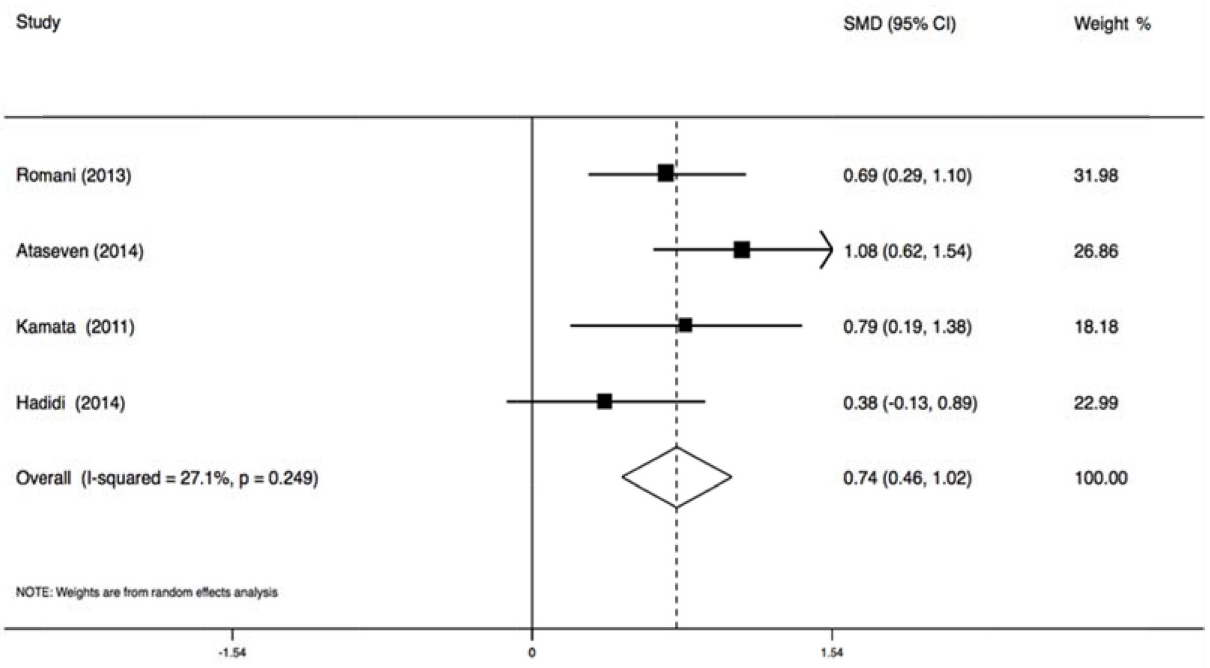

Supplementary Figure 19: Forest plot of the differences in serum levels of lipocalin-2 between psoriasis patients and healthy controls.

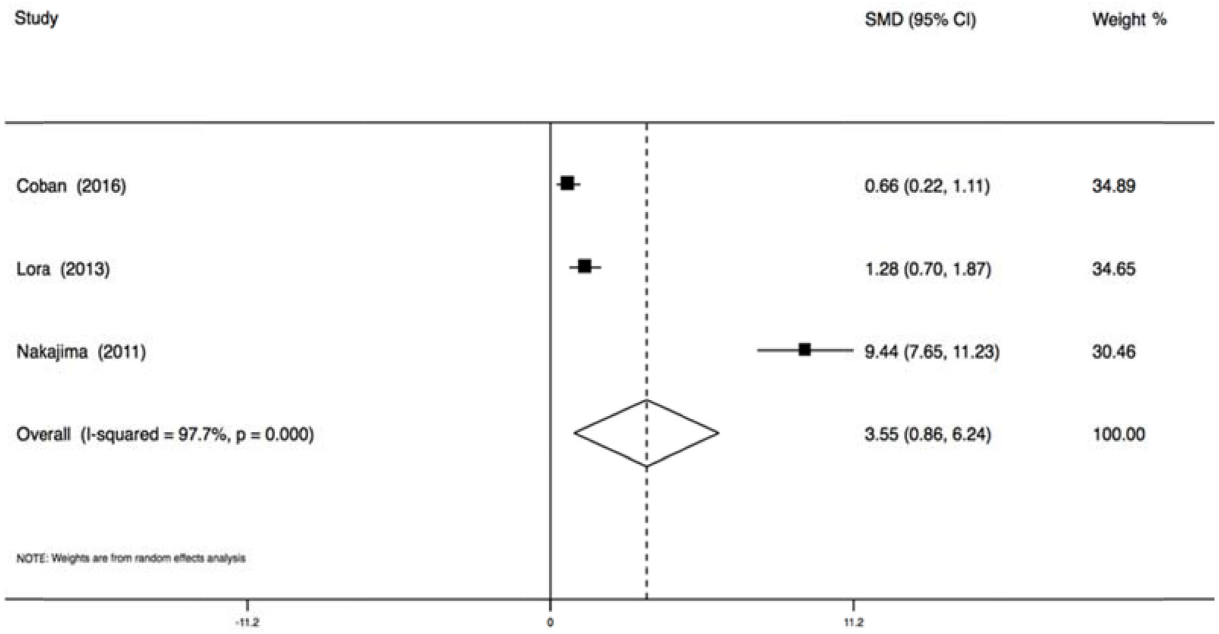

Supplementary Figure 20: Forest plot of the differences in serum levels of chemerin between psoriasis patients and healthy controls.

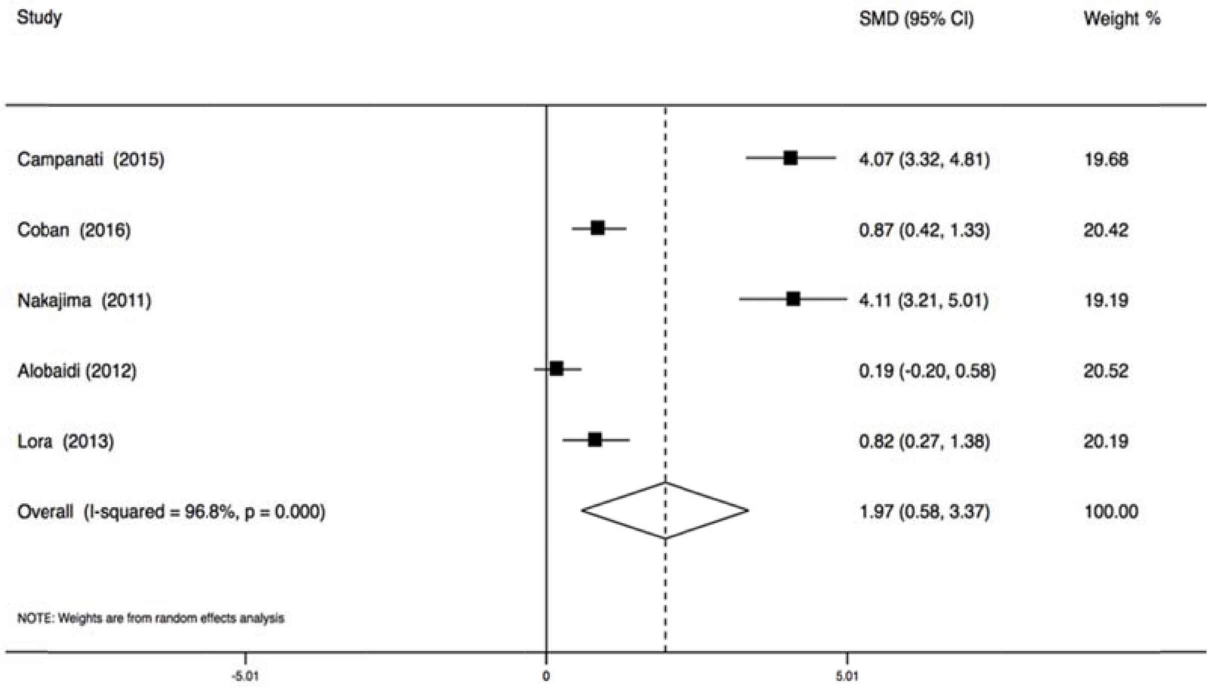

Supplementary Figure 21: Forest plot of the differences in serum levels of resistin between psoriasis patients and healthy controls.

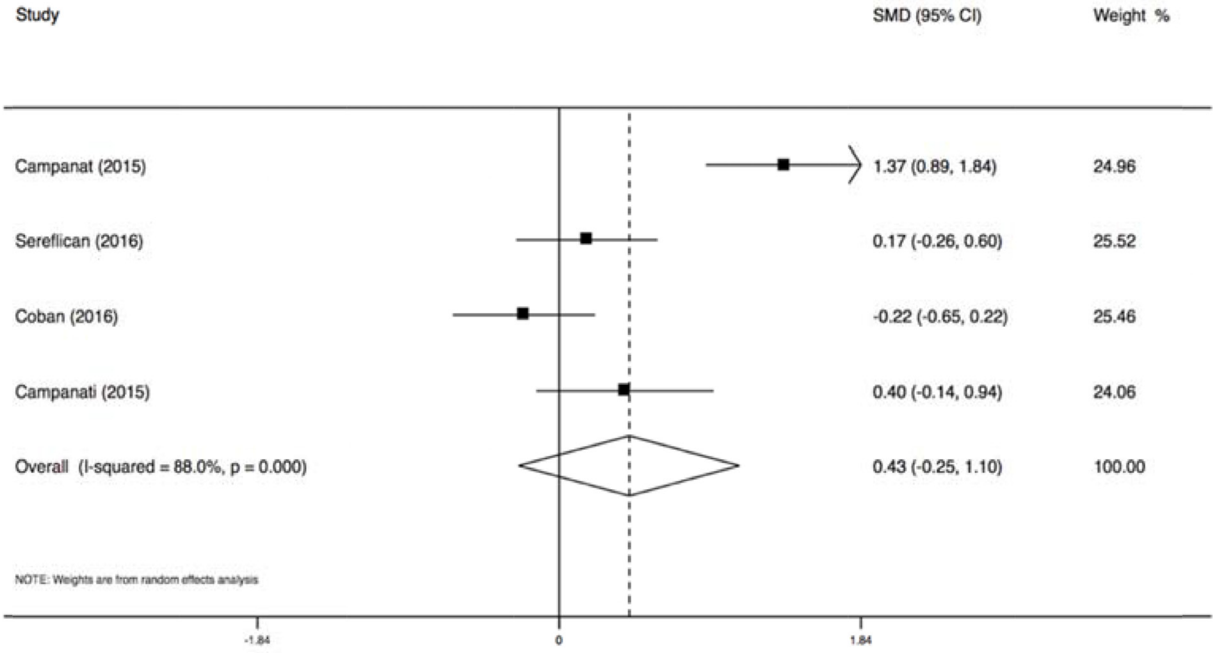

Supplementary Figure 22: Forest plot of the differences in serum levels of visfatin between psoriasis patients and healthy controls.

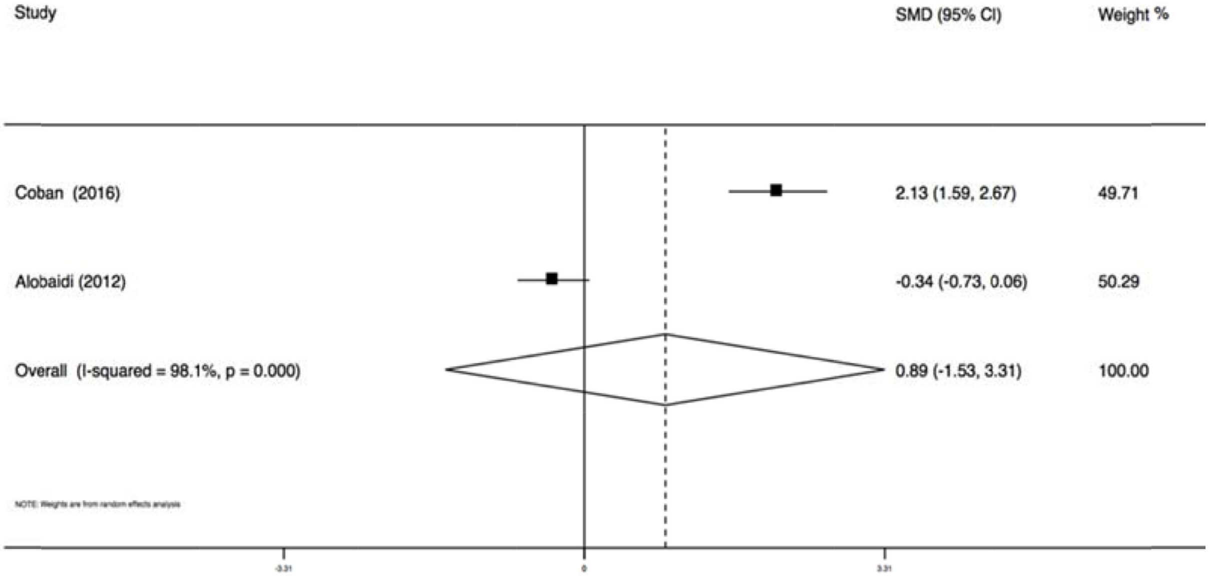

Supplementary Figure 23: Forest plot of the differences in serum levels of omentin between psoriasis patients and healthy controls.

Supplementary Table 1: Stratified meta-analysis of all studied serum markers and psoriasis. See Supplementary_Table 1

Supplementary Table 2: Characteristics of the included studies. See Supplementary_Table 2

Supplementary Table 3: Summary of pooled standardized mean differences (SMDs), and meta-regression analyses for all studied serum markers. See Supplementary_Table 3
