# Supplementary material for: Serum levels of adipokines and cytokines in psoriasis patients: a systematic review and meta-analysis
Source: Oncotarget. 2017 Nov 1;9(1):1266–78. doi: 10.18632/oncotarget.22260 (PMC5787437; doi:10.18632/oncotarget.22260)
Supplement: Supplementary file 2 [file oncotarget-09-1266-s002.docx]

**Supplementary Table 1: Stratified meta-analysis of all studied serum markers and psoriasis**

| Subgroup | N | SMD | Lower | Upper | I^2^ |
| --- | --- | --- | --- | --- | --- |
| TNFα |  |  |  |  |  |
| Caucasians | 9 | 1.43 | 0.70 | 2.17 | 93.90% |
| Asians | 8 | 1.25 | 0.42 | 2.09 | 94.80% |
| Case control | 15 | 1.39 | 0.81 | 1.98 | 94.70% |
| Cross-sectional | 2 | 1.03 | 0.24 | 1.81 | 62.20% |
| Psoriasis vulgaris | 8 | 1.11 | 0.45 | 1.77 | 91.30% |
| Others* | 9 | 1.57 | 0.72 | 2.42 | 94.80% |
| IFN-γ |  |  |  |  |  |
| Caucasians | 4 | 1.32 | 0.09 | 2.56 | 88.70% |
| Asians | 6 | 1.78 | 0.16 | 3.39 | 97.90% |
| Case control | 10 | 1.84 | 0.70 | 2.97 | 96.90% |
| Cross-sectional | 0 | NA |  |  |  |
| Psoriasis vulgaris | 6 | 0.93 | 0.22 | 1.65 | 83.90% |
| Others | 4 | 2.30 | 0.01 | 4.59 | 98.4% |
| IL-1β |  |  |  |  |  |
| Caucasians | 2 | 0.17 | -0.29 | 0.62 | 5.10% |
| Asians | 3 | -0.04 | -1.10 | 1.03 | 90.90% |
| Case control | 3 | -0.04 | -1.10 | 1.03 | 90.90% |
| Cross-sectional | 2 | 0.17 | -0.29 | 0.62 | 5.10% |
| Psoriasis vulgaris | 4 | -0.05 | -0.80 | 0.70 | 85.70% |
| Others | 1 | 0.53 | -0.14 | 1.20 | 0 |
| IL-2 |  |  |  |  |  |
| Caucasians | 2 | 1.07 | 0.21 | 1.93 | 79.20% |
| Asians | 3 | 0.59 | 0.09 | 1.09 | 67.50% |
| Case control | 5 | 0.78 | 0.36 | 1.19 | 70.80% |
| Cross-sectional | 0 | NA |  |  |  |
| Psoriasis vulgaris | 2 | 0.57 | 0.16 | 0.98 | 0.00% |
| Others | 3 | 0.89 | 0.24 | 1.52 | 81.9% |
| IL-4 |  |  |  |  |  |
| Caucasians | 1 | -0.11 | -0.89 | 0.66 | NA |
| Asians | 1 | 0.40 | -0.04 | 0.83 | NA |
| Case control | 2 | 1.32 | 0.69 | 1.95 | 95.00% |
| Cross-sectional | 0 | NA |  |  |  |
| Psoriasis vulgaris | 2 | 0.25 | -0.21 | 0.70 | 20.80% |
| Others | 0 | NA |  |  |  |
| IL-6 |  |  |  |  |  |
| Caucasians | 8 | 0.81 | 0.31 | 1.30 | 87.40% |
| Asians | 6 | 2.04 | 0.61 | 3.47 | 97.00% |
| Case control | 14 | 1.32 | 0.69 | 1.95 | 95.00% |
| Cross-sectional | 0 | NA |  |  |  |
| Psoriasis vulgaris | 6 | 1.11 | 0.11 | 2.10 | 95.00% |
| Others | 8 | 1.49 | 0.61 | 2.37 | 95.6% |
| IL-8 |  |  |  |  |  |
| Caucasians | 6 | 1.46 | 0.67 | 2.26 | 88.50% |
| Asians | 4 | 1.61 | 0.25 | 2.97 | 95.70% |
| Case control | 9 | 1.75 | 0.98 | 2.52 | 93.10% |
| Cross-sectional | 1 | 0.36 | -0.20 | 0.91 | NA |
| Psoriasis vulgaris | 6 | 1.41 | 0.50 | 2.32 | 89.60% |
| Others | 4 | 1.77 | 0.65 | 2.90 | 94.90% |
| IL-17 |  |  |  |  |  |
| Caucasians | 4 | 0.27 | 0.04 | 0.49 | 0.00% |
| Asians | 7 | 0.54 | -0.44 | 1.51 | 96.10% |
| Case control | 11 | 0.44 | -0.16 | 1.03 | 94.00% |
| Cross-sectional | 0 | NA |  |  |  |
| Psoriasis vulgaris | 5 | -0.10 | -0.56 | 0.36 | 75.20% |
| Others | 8 | 0.59 | -0.17 | 1.35 | 94.90% |
| IL-18 |  |  |  |  |  |
| Caucasians | 2 | 1.46 | 0.69 | 2.23 | 59.20% |
| Asians | 1 | 1.76 | 1.43 | 2.09 | NA |
| Case control | 3 | 1.62 | 1.22 | 2.03 | 38.70% |
| Cross-sectional | 0 | NA |  |  |  |
| Psoriasis vulgaris | 1 | 1.76 | 1.43 | 2.09 | NA |
| Others | 2 | 1.48 | 0.80 | 2.17 | 66.60% |
| IL-21 |  |  |  |  |  |
| Caucasians | 0 | NA |  |  |  |
| Asians | 2 | 1.55 | -0.21 | 3.32 | 94.60% |
| Case control | 2 | 1.55 | -0.21 | 3.32 | 94.60% |
| Cross-sectional | 0 | NA |  |  |  |
| Psoriasis vulgaris | 2 | 1.55 | -0.21 | 3.32 | 94.60% |
| Others | 0 | NA |  |  |  |
| IL-22 |  |  |  |  |  |
| Caucasians | 2 | 0.87 | 0.18 | 1.56 | 67.00% |
| Asians | 4 | 0.82 | -0.15 | 1.78 | 91.30% |
| Case control | 6 | 0.84 | 0.21 | 1.46 | 86.80% |
| Cross-sectional | 0 | NA |  |  |  |
| Psoriasis vulgaris | 3 | 0.57 | -0.11 | 1.24 | 78.00% |
| Others | 3 | 1.10 | -0.07 | 2.27 | 91.6% |
| IL-23 |  |  |  |  |  |
| Caucasians | 3 | 0.89 | -0.85 | 2.63 | 97.60% |
| Asians | 3 | 0.42 | -0.31 | 1.15 | 78.50% |
| Case control | 6 | 0.66 | -0.25 | 1.58 | 94.70% |
| Cross-sectional | 0 | NA |  |  |  |
| Psoriasis vulgaris | 3 | 1.26 | -0.14 | 2.66 | 95.60% |
| Others | 3 | 0.34 | -0.25 | 0.85 | 85.30% |
| sE-selectin |  |  |  |  |  |
| Caucasians | 5 | 2.03 | 0.86 | 3.21 | 90.76% |
| Asians | 2 | 1.78 | 0.31 | 3.24 | 92.80% |
| Case control | 6 | 1.90 | 1.07 | 2.74 | 90.50% |
| Cross-sectional | 0 | NA |  |  |  |
| Psoriasis vulgaris | 4 | 2.50 | 1.24 | 3.74 | 90.70% |
| Others | 2 | 0.98 | 0.65 | 1.31 | 0% |
| IL-10 |  |  |  |  |  |
| Caucasians | 3 | 0.63 | -0.73 | 1.99 | 94.60% |
| Asians | 4 | -1.45 | -4.20 | 1.31 | 99.00% |
| Case control | 7 | -0.56 | -2.22 | 1.10 | 98.50% |
| Cross-sectional | 0 | NA |  |  |  |
| Psoriasis vulgaris | 5 | 0.44 | -0.39 | 1.27 | 91.60% |
| Others | 2 | -3.05 | -9.83 | 3.74 | 99.6% |
| IL-12 |  |  |  |  |  |
| Caucasians | 4 | -0.53 | -1.68 | 0.62 | 91.60% |
| Asians | 3 | 0.99 | 0.36 | 1.62 | 78.10% |
| Case control | 7 | 0.22 | -0.55 | 1.00 | 92.50% |
| Cross-sectional | 0 | NA |  |  |  |
| Psoriasis vulgaris | 4 | -0.29 | -1.54 | 0.96 | 91.70% |
| Others | 3 | 0.58 | -0.59 | 1.76 | 95.0% |
| Adiponectin |  |  |  |  |  |
| Caucasians | 7 | -1.17 | -2.22 | -0.12 | 97.30% |
| Asians | 2 | 0.03 | -1.64 | 1.69 | 94.10% |
| Case control | 9 | -0.90 | -1.78 | -0.02 | 96.70% |
| Cross-sectional | 0 | NA |  |  |  |
| Psoriasis vulgaris | 6 | -1.29 | -2.55 | -0.03 | 97.70% |
| Others | 3 | -0.15 | -1.14 | 0.84 | 90.7% |
| Omentin |  |  |  |  |  |
| Caucasians | 2 | 0.89 | -1.53 | 3.31 | 98.10% |
| Asians | 0 | NA |  |  |  |
| Case control | 2 | 0.89 | -1.53 | 3.31 | 98.10% |
| Cross-sectional | 0 | NA |  |  |  |
| Psoriasis vulgaris | 2 | 0.89 | -1.53 | 3.31 | 98.10% |
| Others | 0 | NA | 0 | NA | 0 |
| Chemerin |  |  |  |  |  |
| Caucasians | 2 | 0.94 | 0.34 | 1.55 | 63.50% |
| Asians | 1 | 9.44 | 7.65 | 11.23 | NA |
| Case control | 3 | 3.55 | 0.87 | 6.24 | 97.70% |
| Cross-sectional | 0 | NA |  |  |  |
| Psoriasis vulgaris | 2 | 0.94 | 0.34 | 1.55 | 63.50% |
| Others | 1 | 9.44 | 7.65 | 11.23 | NA |
| Visfatin |  |  |  |  |  |
| Caucasians | 4 | 0.43 | -0.25 | 1.10 | 88.00% |
| Asians | 0 | NA |  |  |  |
| Case control | 4 | 0.43 | -0.25 | 1.10 | 88.00% |
| Cross-sectional | 0 | NA |  |  |  |
| Psoriasis vulgaris | 3 | 0.515 | -0.435 | 1.465 | 91.50% |
| Others | 1 | 0.168 | -0.260 | 0.597 | NA |
| Lipocalin-2 |  |  |  |  |  |
| Caucasians | 3 | 0.85 | 0.58 | 1.12 | 0.00% |
| Asians | 1 | 0.38 | -0.13 | 0.89 | NA |
| Case control | 4 | 0.74 | 0.46 | 1.02 | 27.10% |
| Cross-sectional | 0 | NA |  |  |  |
| Psoriasis vulgaris | 1 | 0.38 | -0.13 | 0.89 | NA |
| Others | 3 | 0.85 | 0.58 | 1.12 | 0.00% |
| C3 |  |  |  |  |  |
| Caucasians | 2 | 0.89 | 0.25 | 1.54 | 83.20% |
| Asians | 1 | 0.00 | -0.20 | 0.20 | NA |
| Case control | 2 | 0.67 | -0.43 | 1.76 | 95.20% |
| Cross-sectional | 1 | 0.58 | 0.28 | 0.88 | NA |
| Psoriasis vulgaris | 1 | 0.58 | 0.28 | 0.88 | NA |
| Others | 2 | 0.67 | -0.43 | 1.76 | 95.20% |
| Fibrinogen |  |  |  |  |  |
| Caucasians | 9 | 0.98 | 0.53 | 1.42 | 89.30% |
| Asians | 0 | NA |  |  |  |
| Case control | 10 | 1.05 | 0.61 | 1.48 | 89.70% |
| Cross-sectional | 0 | NA |  |  |  |
| Psoriasis vulgaris | 4 | 0.47 | 0.09 | 0.85 | 76.00% |
| Others | 5 | 1.40 | 0.99 | 1.81 | 61.50% |
| Resistin |  |  |  |  |  |
| Caucasians | 4 | 1.46 | 0.12 | 2.79 | 96.30% |
| Asians | 1 | 4.11 | 3.21 | 5.02 | NA |
| Case control | 5 | 1.97 | 0.58 | 3.37 | 96.80% |
| Cross-sectional | 0 | NA |  |  |  |
| Psoriasis vulgaris | 4 | 1.46 | 0.12 | 2.79 | 96.30% |
| Others | 1 | 4.11 | 3.21 | 5.02 | NA |

C3, complement 3; CI, confidence interval; IFN, interferon; NA, not available; IL, interleukin; sE-selectin, soluble E-selectin; SMD, standardized mean difference; TNF, tumour necrosis factor.

*”Others” does not include the plaque psoriasis.
