# Supplementary material for: Serum levels of adipokines and cytokines in psoriasis patients: a systematic review and meta-analysis
Source: Oncotarget. 2017 Nov 1;9(1):1266–78. doi: 10.18632/oncotarget.22260 (PMC5787437; doi:10.18632/oncotarget.22260)
Supplement: Supplementary file 3 [file oncotarget-09-1266-s003.docx]

**Supplementary Table 2: Characteristics of the included studies**

| Author | Year | Country | Psoriasis group | | | | | | Control group | | | | Markers measurement methods | Study quality^＊^ |
| --- | --- | --- | --- | --- | --- | --- | --- | --- | --- | --- | --- | --- | --- | --- |
|  |  |  | PASI | | N | Age | | Male(N) | N | Age | | Male(N) |  |  |
|  |  |  | Mean | SD |  | Mean | SD |  |  | Mean | SD |  |  |  |
| Takahashi^8^ | 2009 | Japan | 7.3 | 4.2 | 122 | 47.5 | 7.6 | 81 | 78 | 38.6 | 7.2 | 54 | ELISA | 6 |
| Choe^9^ | 2012 | Korea | 7.9 | 5.2 | 71 | 36.3 | 14.0 | 41 | 15 | 34.2 | 12.6 | NP | NO | 7 |
| Alshorafa^10^ | 2012 | China | 11.9 | 5 | 15 | 35.5 | 19.0 | 6 | 15 | 33.4 | 20.8 | 8 | ELISA | 6 |
| Z. He^11^ | 2012 | China | 8.1 | 6 | 37 | 31.2 | 12.0 | 22 | 37 | 31.1 | 10.6 | 22 | ELISA | 5 |
| Kyriakou^12^ | 2014 | Greece | 5.2 | 4 | 32 | 44.5 | 16.0 | 9 | 32 | NP | NP | NP | NO | 6 |
| Baran^13^ | 2015 | Poland | 16.5 | 1 | 49 | 48.6 | 2.4 | 34 | 16 | NP | NP | NP | ELISA | 7 |
| Campanati^14^ | 2015 | Italy | 19.9 | 4 | 47 | 52.0 | 11.0 | 27 | 39 | NP | NP | 20 | NO | 7 |
| Fotiadou^15^ | 2015 | Greece | 16.1 | 7 | 35 | 47.0 | 16.0 | 28 | 20 | 48.5 | 14.2 | 15 | ELISA | NA |
| Hayashi^16^ | 2015 | Japan | NP | NP | 31 | NP | NP | NP | 26 | NP | NP | NP | ELISA | 6 |
| Bilgic^17^ | 2015 | Turkey | 12.0 | 9 | 45 | 35.8 | 12.0 | 22 | 43 | 33.6 | 9.5 | 23 | ELISA | 7 |
| Xuan^18^ | 2015 | China | 12.8 | 8 | 62 | 36.2 | 11.0 | 42 | 20 | 37.3 | 12.9 | 12 | ELISA | 4 |
| Sereflican^19^ | 2016 | Turkey | 6.1 | 4 | 42 | 35.7 | 12.0 | 23 | 42 | 33.9 | 11.2 | 19 | ELISA | 6 |
| Coban^20^ | 2016 | Turkey | 5.8 | 2 | 35 | 44.4 | 12.0 | 25 | 50 | 40.5 | 13.5 | 32 | NO | 6 |
| Yilmaz^21^ | 2012 | Turkey | 6.2 | 5.6 | 70 | 39.9 | 14.9 | 36 | 50 | 39.7 | 13.4 | 24 | ELISA | 6 |
| Akcali^22^ | 2013 | Turkey | 20.3 | 6 | 50 | 38.6 | 13.0 | 26 | 40 | 40.5 | 14.6 | 20 | NO | 6 |
| Torres^23^ | 2014 | Portugal | 15.4 | 7 | 80 | 48.1 | 11.0 | 52 | 95 | 48.4 | 11.9 | 41 | NO | NA |
| Gkalpakiotis^24^ | 2016 | Portugal | 26.7 | 8 | 21 | 43.0 | 9.1 | 9 | 21 | 42.3 | 9.4 | 9 | ELISA | 7 |
| Nam^25^ | 2015 | Korea | 8.2 | 4 | 68 | 36.6 | 14.2 | NP | 10 | 32.6 | 10.0 | NP | ELISA | 6 |
| Zhou^26^ | 2016 | China | 15.7 | 7 | 214 | 41.0 | 13.0 | 159 | 165 | 42.2 | 9.7 | 109 | NO | 6 |
| Dogan^27^ | 2014 | Turkey | NP | NP | 30 | 34.1 | 6.7 | 15 | 30 | 31.9 | 6.5 | 15 | NO | 7 |
| Gerdes^28^ | 2011 | Germany | 14.3 | 12.6 | 79 | 47.7 | 1.5 | 40 | 80 | 48.0 | 1.4 | 41 | ELISA | 7 |
| Johnston^29^ | 2008 | Iceland | 15.3 | 10 | 30 | 52.9 | 23.0 | 16 | 29 | 47.1 | 24.2 | 13 | ELISA | 7 |
| Shibata^30^ | 2009 | Japan | NP | NP | 23 | 52.9 | 13.0 | 23 | 22 | 49.7 | 12.5 | NP | ELISA | 6 |
| Kaur^31^ | 2011 | Estonia | 10.1 | 7 | 60 | 41.0 | 14.0 | 39 | 48 | 41.0 | 7.0 | 24 | ELISA | 7 |
| Nakajima^32^ | 2011 | Japan | NP | NP | 30 | NP | NP | NP | 30 | NP | NP | NP | NO | 7 |
| Alobaidi^33^ | 2012 | Iraq | NP | NP | 50 | NP | NP | 21 | 50 | NP | NP | 21 | ELISA | 6 |
| Romani^34^ | 2013 | Spain | 15.6 | 5 | 50 | 46.4 | 17.0 | 31 | 50 | 46.1 | 17.5 | 31 | ELISA | 6 |
| Lora^35^ | 2013 | Italy | 13.3 | 12 | 27 | 49.9 | 12.3 | 15 | 27 | 48.1 | 8.2 | 15 | ELISA | 6 |
| Ataseven^36^ | 2014 | *urkey* | 8.3 | 7 | 56 | 39.8 | 18.0 | 21 | 33 | 36.2 | 14.2 | 11 | ELISA | 7 |
| Elango^37^ | 2012 | India | NP | NP | 22 | 42.9 | 13.0 | NP | 20 | 45.1 | 11.3 | NP | ELISA | 5 |
| Murakami^38^ | 2011 | Japan | NP | NP | 48 | NP | NP | NP | 20 | NP | NP | NP | ELISA | 7 |
| Michalak-Stoma^39^ | 2013 | Poland | 15.7 | 10 | 60 | NP | NP | 50 | 30 | NP | NP | NP | ELISA | 6 |
| Zhang^40^ | 2010 | China | NP | NP | 54 | NP | NP | NP | 18 | NP | NP | NP | ELISA | 5 |
| Jensen^41^ | 2011 | Denmark | 7.3 | 4 | 30 | 44.3 | 18.4 | 18 | 30 | 45.0 | 11.7 | 15 | NO | 7 |
| Abdel-hamid^42^ | 2011 | Egypt | 9.3 | 5 | 60 | 40.2 | 17.0 | 29 | 21 | 43.4 | 15.4 | 10 | ELISA | 7 |
| Kural ^43^ | 2003 | Turkey | 5.8 | 5 | 35 | NP | NP | 17 | 35 | NP | NP | 19 | NO | 6 |
| Anderson^44^ | 2010 | Boston | 8.5 | 4 | 14 | NP | NP | NP | 14 | NP | NP | NP | NO | NA |
| Gisondi ^45^ | 2010 | Verona | 10.3 | 9 | 172 | 57.3 | 15.0 | 119 | 198 | 56.1 | 21.2 | 122 | NO | 7 |
| Arican^46^ | 2005 | Turkey | 9.3 | 8 | 30 | 35.0 | 16.0 | 18 | 23 | NP | NP | NP | ELISA | 6 |
| Kamata^47^ | 2011 | Japan | 8.77 | 2 | 37 | 56.1 | 14.0 | 27 | 17 | 38.6 | 10.9 | 12 | ELISA | 7 |
| Arias-santiago^48^ | 2012 | Spain | NP | NP | 72 | NP | NP | NP | 61 | NP | NP | NP | NO | 6 |
| Alpsoy^49^ | 2014 | Turkey | 10.5 | 7 | 60 | 47.7 | 13.0 | 29 | 50 | 44.6 | 11.3 | 24 | NO | 6 |
| Balta^50^ | 2013 | Turkey | 10.8 | 6 | 29 | 38 5 | 13 6 | 16 | 35 | 36 4 | 11 7 | 3 | ELISA | 6 |
| Hadidi^51^ | 2014 | Egypt | 15.03 | 3 | 30 | 48.4 | 12.0 | 16 | 30 | 47.1 | 10.5 | 16 | ELISA | 6 |
| Borska^52^ | 2006 | Prague | NP | NP | 56 | NP | NP | NP | 55 | NP | NP | NP | ELISA | 6 |
| Czech^53^ | 1996 | Germany | NP | NP | 16 | NP | NP | 9 | 16 | NP | NP | 8 | ELISA | 6 |
| Pitae^54^ | 1999 | Italy | 5.4 | 3 | 24 | NP | NP | NP | 20 | NP | NP | NP | ELISA | 7 |
| Gangemi^55^ | 2003 | Italy | NP | NP | 16 | 39.9 | 16.0 |  | 16 | 41.8 | 16.6 |  | ELISA | 6 |
| Jacob^56^ | 2003 | USA | NP | NP | 12 | NP | NP | NP | 5 | NP | NP | NP | ELISA | 6 |
| Kanda^57^ | 2010 | Japan | 8.1 | 7 | 61 | 51.9 | 21.0 | 45 | 31 | 46.4 | 15.0 | 20 | ELISA | 6 |
| Jadali^58^ | 2007 | Iran | 6.3 | 6 | 40 | 38.3 | 14.0 | 18 | 40 | 38.7 | 11.8 | 18 | ELISA | 6 |
| Karabudak^59^ | 2008 | *Turkey* | 13 | 7 | 20 | 23.0 | 4.0 | NP | 20 | 21.0 | 1.0 | NP | NO | 6 |
| Laurent^60^ | 1981 | London | NP | NP | 15 | NP | NP | NP | 21 | NP | NP | NP | NO | 6 |
| Long^61^ | 2010 | China | 23.7 | 10 | 58 | NP | NP | 36 | 50 | NP | NP | NP | NO | 7 |
| Qiu^62^ | 2005 | China | NP | NP | 33 | NP | NP | NP | 30 | NP | NP | NP | NO | 5 |
| Pereira^63^ | 2004 | Portugal | NP | NP | 60 | 46.0 | 12.0 | 24 | 40 | 47.0 | 13.0 | 22 | NO | 6 |
| Szegedi^64^ | 2003 | Hungary | 20.1 | 4.5 | 18 | 47.2 | 17.6 | 14 | 10 | 33.7 | 4.8 | 6 | ELISA | 7 |
| Szepietowski^65^ | 1998 | Poland | 20.7 | 8 | 33 | 38.3 | 39.0 | 21 | 10 | NP | NP | NP | ELISA | 6 |
| Szepietowski^66^ | 2000 | Poland | 26 | 11 | 40 | 46.5 | 16.9 | NP | 18 | NP | NP | NP | ELISA | 7 |
| Toruniowa^67^ | 1995 | Poland | NP | NP | 20 | 60.8 | 13.0 | NP | 14 | 41.2 | 9.6 | NP | NO | 7 |
| Kural^68^ | 2003 | Turkey | 5.5 | 4 | 30 | NP | NP | 13 | 30 | NP | NP | 15 | ELISA | 6 |
| Kitamura^69^ | 1999 | Japan | NP | NP | 30 | NP | NP | 15 | 20 | NP | NP | 10 | ELISA | 7 |
| Biasa^70^ | 1998 | Italy | NP | NP | 15 | 43.4 | 11.0 | 4 | 22 | NP | NP | NP | ELISA | 7 |

ELISA, enzyme linked immunosorbent assay; N, number of individuals; NA, not available; NO, not ELISA; NP, not reported; SD, standard deviation.

^＊^Study quality was assessed by Newcastle-Ottawa Scale.
