# Supplementary material for: Serum levels of adipokines and cytokines in psoriasis patients: a systematic review and meta-analysis
Source: Oncotarget. 2017 Nov 1;9(1):1266–78. doi: 10.18632/oncotarget.22260 (PMC5787437; doi:10.18632/oncotarget.22260)
Supplement: Supplementary file 4 [file oncotarget-09-1266-s004.docx]

**Supplementary Table 3: Summary of pooled standardized mean differences (SMDs), and meta-regression analyses for all studied serum markers**

| Biomarker | N | SMD | Lower | Upper | I^2^ | Begger | Egger | Meta-regression analyses^*^, regression coefficient (95% CI) | | | | | | | | | | | | | | | | | | |
| --- | --- | --- | --- | --- | --- | --- | --- | --- | --- | --- | --- | --- | --- | --- | --- | --- | --- | --- | --- | --- | --- | --- | --- | --- | --- | --- |
|  |  |  |  |  |  |  |  | Age | | | | | Sex | | | | PASI | | | | Quality scores | | | | | |
|  |  |  |  |  |  |  |  | Slope | Lower | Upper | | P | Slope | Lower | Upper | P | Slope | Lower | Upper | P | Slope | Lower | | Upper | | P |
| Pro-inflammatory cytokines | | | | | | | | | | | | | | | | | | | | | | | | | | |
| TNF-α | 17 | 1.35 | 0.82 | 1.88 | 94.00% | 0.76 | 0.34 | -0.07 | -0.43 | | 0.30 | 0.60 | 1.41 | -16.27 | 19.09 | 0.82 | 0.00 | -0.55 | 0.56 | 0.99 | 0.42 | | -2.19 | | 3.02 | 0.64 |
| IFN-γ | 10 | 1.84 | 0.70 | 2.97 | 96.90% | 0.24 | 0.31 | 0.09 | -0.16 | | 0.35 | 0.41 | -10.12 | -48.94 | 28.69 | 0.38 | -0.10 | -0.57 | 0.37 | 0.62 | -0.87 | | -6.50 | | 4.75 | 0.73 |
| IL-1β | 5 | 0.06 | -0.60 | 0.71 | 84.00% | 0.22 | 0.50 | -0.09 | -0.20 | | 0.02 | 0.17 | NA |  |  |  | -0.01 | -0.36 | 0.34 | 0.94 | 0.71 | | -16.34 | | 17.75 | 0.69 |
| IL-2 | 5 | 0.78 | 0.36 | 1.19 | 70.80% | 0.53 | 0.26 | 0.04 | -0.27 | | 0.35 | 0.67 | NA |  |  |  | -0.23 | -0.96 | 0.51 | 0.40 | -0.28 | | -2.36 | | 1.79 | 0.69 |
| IL-4 | 2 | 0.25 | -0.21 | 0.70 | 20.80% | 0.42 | 0.75 | NA |  | |  |  | NA |  |  |  | NA |  |  |  | NA | |  | |  |  |
| IL-6 | 14 | 1.32 | 0.69 | 1.95 | 95.00% | 0.55 | 0.18 | 0.24 | -0.23 | | 0.71 | 0.16 | -18.25 | -41.30 | 4.80 | 0.08 | -0.11 | -0.51 | 0.30 | 0.38 | 2.70 | | -2.10 | | 7.50 | 0.14 |
| IL-8 | 10 | 1.59 | 0.87 | 2.31 | 93.00% | 0.52 | 0.43 | 0.18 | -0.50 | | 0.85 | 0.38 | -5.96 | -16.49 | 4.57 | 0.17 | 0.23 | -1.88 | 2.34 | 0.69 | 0.13 | | -3.30 | | 3.55 | 0.89 |
| IL-12 | 7 | 0.22 | -0.55 | 1.00 | 92.50% | 0.41 | 0.67 | 0.14 | -0.23 | | 0.50 | 0.13 | NA |  |  |  | 0.49 | -0.92 | 1.90 | 0.14 | 0.61 | | -3.95 | | 5.18 | 0.34 |
| IL-17 | 11 | 0.44 | -0.16 | 1.03 | 94.00% | 0.39 | 0.08 | 0.02 | -0.19 | | 0.24 | 0.77 | -17.34 | -26.60 | -8.07 | 0.02 | -0.11 | -0.97 | 0.76 | 0.75 | 0.24 | | -1.93 | | 2.40 | 0.77 |
| IL-18 | 3 | 1.62 | 1.22 | 2.03 | 38.70% | 0.93 | 0.24 | NA |  | |  |  | NA |  |  |  | NA |  |  |  | NA | |  | |  |  |
| IL-21 | 2 | 1.55 | -0.21 | 3.32 | 94.60% | NA | NA | NA |  | |  |  | NA |  |  |  | NA |  |  |  | NA | |  | |  |  |
| IL-22 | 6 | 0.84 | 0.21 | 1.46 | 86.80% | 0.27 | 0.90 | -0.02 | -0.22 | | 0.17 | 0.69 | NA |  |  |  | 0.05 | -0.01 | 0.11 | 0.07 | 1.03 | | -0.54 | | 2.61 | 0.14 |
| IL-23 | 6 | 0.66 | -0.25 | 1.58 | 94.70% | 0.15 | 0.17 | 0.53 | -5.48 | | 6.54 | 0.46 | NA |  |  |  | -0.16 | -0.39 | 0.06 | 0.09 | -0.79 | | -3.52 | | 1.93 | 0.47 |
| Anti-inflammatory cytokines | | | | | | | | | | | | | | | | | | | | | | | | | | |
| IL-10 | 7 | -0.56 | -2.22 | 1.10 | 98.50% | 0.87 | 0.12 | -0.13 | -0.93 | | 0.66 | 0.63 | 6.43 | -35.28 | 48.15 | 0.66 | 0.06 | -0.90 | 1.02 | 0.85 | 0.99 | | -7.23 | | 9.21 | 0.77 |
| Adipokines |  |  |  |  |  |  |  |  |  | |  |  |  |  |  |  |  |  |  |  |  | |  | |  |  |
| Adiponectin | 9 | -0.90 | -1.78 | -0.02 | 96.70% | 0.23 | 0.35 | -0.01 | -0.29 | | 0.28 | 0.96 | -4.61 | -23.16 | 13.94 | 0.53 | -0.11 | -0.46 | 0.23 | 0.41 | 0.02 | | -2.75 | | 2.78 | 0.99 |
| Omentin | 2 | 0.89 | -1.53 | 3.31 | 98.10% | NA | NA | NA |  | |  |  | NA |  |  |  | NA |  |  |  | NA | |  | |  |  |
| Chemerin | 3 | 3.55 | 0.87 | 6.24 | 97.70% | 0.54 | 0.17 | NA |  | |  |  | NA |  |  |  | NA |  |  |  | 8.50 | | -4.54 | | 21.54 | 0.12 |
| Visfatin | 4 | 0.43 | -0.25 | 1.10 | 88.00% | 0.32 | 0.78 | 0.05 | -0.16 | | 0.27 | 0.42 | -3.29 | -46.92 | 40.33 | 0.78 | 0.07 | -0.09 | 0.22 | 0.21 | 1.27 | | -0.26 | | 2.81 | 0.17 |
| Lipocalin-2 | 4 | 0.74 | 0.46 | 1.02 | 27.10% | 0.75 | 0.62 | -0.02 | -0.14 | | 0.10 | 0.50 | 4.04 | -5.74 | 13.83 | 0.22 | -0.06 | -0.21 | 0.10 | 0.25 | 0.40 | | -0.66 | | 1.46 | 0.25 |
| Resistin | 5 | 1.97 | 0.58 | 3.37 | 96.80% | 0.14 | 0.81 | 0.33 | -3.93 | | 4.59 | 0.51 | 16.87 | -40.98 | 74.72 | 0.34 | 0.22 | -1.60 | 2.04 | 0.37 | 1.49 | | -0.17 | | 2.80 | 0.23 |
| Other inflammatory markers | | | | | | | | | | | | | | | | | | | | | | | | | | |
| sE-selectin | 6 | 1.90 | 1.70 | 2.74 | 90.50% | 0.18 | 0.44 | NA |  | |  |  | NA |  |  |  | 0.08 | -1.31 | 1.47 | 0.60 | 0.47 | | -4.19 | | 5.13 | 0.79 |
| Fibrinogen | 9 | 0.98 | 0.53 | 1.42 | 89.30% | 0.44 | 0.27 | -0.05 | -0.23 | | 0.12 | 0.16 | -4.01 | -8.34 | 0.31 | 0.06 | -0.04 | -0.19 | 0.11 | 0.51 | -0.81 | | -1.67 | | 0.45 | 0.06 |
| C3 | 3 | 0.62 | 0.03 | 1.21 | 91.20% | 0.18 | 0.20 | NA |  | |  |  | NA |  |  |  | NA |  |  |  | NA | |  | |  |  |

C3, complement 3; CI, confidence interval; IFN, interferon; NA, not available. IL, interleukin; PASI, Psoriasis Area and Severity Index; sE-selectin, soluble E-selectin; SMD, standardized mean differences; TNF, tumour necrosis factor.

^*^ Mixed-effects regression. The regression coefficient represents the slope of the regression line.
